# A modified expectation–maximization algorithm for accelerated item response theory model estimation with large datasets

**DOI:** 10.3758/s13428-026-02996-0

**Published:** 2026-04-21

**Authors:** Tianying Feng, Li Cai

**Affiliations:** University of California, Los Angeles, 315 SEIS Building, Los Angeles, CA 90095-1522 USA

**Keywords:** EM algorithm, Item response theory, Large data, Estimation

## Abstract

The expectation–maximization (EM) algorithm is widely used for parameter estimation in item response theory (IRT) modeling. However, when applied to datasets with large numbers of individuals and items, the standard EM algorithm can be slow to converge, with computationally expensive E-steps. We propose a modified EM algorithm to accelerate estimation for unidimensional two-parameter logistic IRT models. The modified algorithm uses a two-stage structure with partial-step updating over data subsets to reduce convergence time, while maintaining comparable accuracy and precision. The first two simulation studies evaluated its performance relative to standard EM, focusing on convergence time, parameter recovery, and standard error estimation across varying subset sizes and item counts. The third study demonstrated its scalability and runtime advantage in a large-scale testing scenario involving one million respondents and 100 items. The fourth study evaluated robustness under departures from unidimensionality. The proposed algorithm showed time reductions under smaller subsets with 36 items, consistent reductions across all subset sizes with 54 items, and the largest reduction (60%) with 40-item forms constructed from 100 items, while maintaining comparable estimation performance. These results highlight the algorithm’s potential for large-scale applications involving tens of thousands of respondents and moderate-to-large item pools, with modifications that can be integrated into existing EM routines. We conclude with directions for operational use and potential extensions to multidimensional IRT estimation.

**Supplementary Information:**

The online version contains supplementary material available at 10.3758/s13428-026-02996-0.

## Introduction

The expectation–maximization (EM) algorithm (Dempster, Laird, & Rubin, 1977) is a widely used tool for estimating item response theory (IRT) models in educational and psychological research. It is an iterative procedure for obtaining maximum likelihood estimates when some data or variables are unobserved. Despite its utility, the EM algorithm can be slow to converge, with diminishing improvements as the parameter iterates approach the solution (Bock & Aitkin, 1981; Baker & Kim, 2004; also see discussant comments in Dempster et al., 1977). The E-step, in particular, becomes computationally expensive as the sample size, the number of items, or the number of latent variables grows.

### Existing extensions on accelerating the EM algorithm

#### E-step variants

Variants that modify the E-step to accelerate convergence have a long history in parallel computing, computational statistics, and machine learning. Early work explored partial E-step formulations (Cappé & Moulines, 2009; Liang & Klein, 2009; Neal & Hinton, 1998; Thiesson, Meek, & Heckerman, 2001). Subsequent studies leveraged distributed and parallel implementations over partitioned data (von Davier, 2016; Yin, Zhang, & Gao, 2018) as well as data restructuring to reduce the number of operations in the E-step (Robitzsch, 2021).

One strategy is to incrementally update the key statistics as the algorithm processes each subset. These algorithms partition the data into disjoint subsets but differ in how they store and update key statistics after each partial E-step. For example, the incremental EM algorithm (Neal & Hinton, 1998) tracks both the overall and subset-level sufficient statistics. With each partial E-step, it updates the overall statistics by subtracting the old subset values and adding the newly computed ones. The stochastic approximation EM algorithm (SAEM; Delyon, Lavielle, & Moulines, 1999; Cappé & Moulines, 2009) uses a weighted interpolation between the current and subset-level statistics, with the weighting controlled by a step size parameter.

Another strategy is fork-and-join parallelization over data partitions within the standard E-step (parallel EM; von Davier, 2016). Under this approach, the E-step is parallelized by subdividing respondents’ data into groups, computing group-specific statistics in parallel, and then aggregating the results. When only the E-step is parallelized, the M-step is performed as in standard EM using the aggregated statistics from the parallel E-step. While both the fork-and-join paradigm and our approach involve data splitting at the implementation level, our focus is on sequential partial updates. We return to this distinction in Section “This paper”.

An alternative strategy restructures the item data into a smaller number of polytomous pseudo-items (Robitzsch, 2021), each representing a pre-specified combination of original items. By pre-computing the response probabilities for these pseudo-items, the approach reduces the number of operations required in the likelihood evaluation step of the E-step. This line of work complements partial E-step strategies by improving computational efficiency through one-time data restructuring. In large-scale settings where low-level implementation details, such as memory allocation and access patterns, can be tightly controlled, this restructuring step could provide substantial practical benefits. In comparison, our focus is on algorithmic modifications to the EM algorithm itself, without restructuring the item data.

#### M-step variants

The M-step of the EM algorithm may also be modified to improve computational efficiency. The generalized EM algorithm (GEM; Dempster et al., 1977) relaxes standard EM’s requirement of maximizing the log-likelihood in the M-step by allowing for a computationally more efficient update that improves the log-likelihood. A subclass of GEM, the expectation-conditional maximization algorithm (ECM; Meng & Rubin, 1993), replaces the M-step of standard EM with multiple conditional maximization steps. As its name suggests, the parallel EM algorithm (von Davier, 2016) can also parallelize the M-step by subdividing response variables into groups. It computes parameter updates in parallel before aggregating them into a global parameter set.

### Why partial-step schemes?

With partial steps, parameters are updated more frequently with reduced computation per pass through the data. This frequent updating allows subsequent steps to leverage more up-to-date parameter estimates, leading to faster convergence. The difference between partial EM and standard EM can be likened to hill climbing. In a partial-step scheme, each iteration of the algorithm takes several noisier but faster and computationally cheaper steps toward a local peak, with each step pointing in the right direction. In contrast, standard EM computes the expected complete data log-likelihood using the whole dataset. It updates the parameters once per iteration, making one more accurate but slower step toward the peak.

While noise from partial updates can help algorithms make rapid progress and escape poor stationary points (e.g., saddle points), excessive noise from stochastic updates can cause the iterates to converge to a "noise ball," wherein they fluctuate around the solution rather than converging to it (Gower, Schmidt, Bach, & Richtarik, 2020). Several techniques can mitigate this issue, including using a sequence of decreasing step sizes that satisfy the Robbins–Monro conditions (Robbins & Monro, 1951), increasing the subset size, computing weighted averages, or incorporating infrequent full-data updates to reduce the variance of subset-driven updates (Chen, Zhu, Teh, & Zhang, 2018; Johnson & Zhang, 2013).

### This paper

In large-scale operational settings, such as national or international testing programs, survey research, or health-related measurement, unidimensional IRT models remain widely used. In these cases, the M-step in EM estimation is analytically tractable and easy to compute, but the E-step becomes the primary computational bottleneck, especially as sample size and item length grow.

We propose a modified EM algorithm that builds on ideas from partial E-steps and generalized M-steps to enable faster estimation of unidimensional IRT models, an area that remains underexplored. Compared to existing E-step variants, the proposed modifications place less emphasis on tuning a step size parameter and its decay schedule, and more on leveraging features of (unidimensional) IRT EM estimation (Section “Features that motivate the proposed modifications”). They exploit data variability to drive computationally cheaper and more frequent sequential partial updates, where subset-specific results feed into subsequent steps, rather than relying on fork-and-join parallelization over data partitions per E-step or M-step. They also augment existing EM routines without altering their core implementation or the original item data.

We organize the remainder of the paper as follows. Section “Background and motivation” introduces the two-parameter logistic IRT model, the standard EM algorithm for unidimensional IRT parameter estimation, and specific components of EM estimation that motivate the proposed modifications. Section “Modified EM algorithm” describes the proposed algorithm that uses a two-stage structure. Section “Simulation studies” presents four simulation studies. The first two studies evaluate the proposed algorithm against standard EM in terms of convergence time, parameter recovery, and standard error estimation under varied data conditions. The third study mimics a large-scale testing scenario, where 1 million respondents are randomly assigned to one of ten 40-item forms, each constructed from a 100-item pool partitioned into blocks. The fourth study serves as a robustness check under messier data conditions (e.g., multidimensionality). Section “Discussion” discusses the findings and concludes with considerations for implementing and improving the proposed algorithm.

## Background and motivation

### Unidimensional two-parameter logistic IRT models

We focus our discussion on unidimensional two-parameter logistic (2PL) IRT models that are not members of the exponential family. Let $\eta$ denote a standard normally distributed latent variable. Let *i* index the items ($i= 1,\dots ,I$), and let $y_{ip}$, coded as 0 or 1, denote Person *p*’s dichotomously scored response to Item *i*. Using $\boldsymbol{\phi }_i$ to collect the two item-specific parameters $a_i$ and $c_i$, the conditional response probabilities are

1 $$\begin{aligned} P_i(\eta _p)&:= P(y_{ip} = 1 \mid \eta _p, \boldsymbol{\phi }_i) = \frac{1}{1+\exp {[-z_{ip}]}}\nonumber \\ &= \frac{1}{1+\exp {[-(a_i \eta _p + c_i)]}} \end{aligned}$$

2 $$\begin{aligned} Q_i(\eta _p)&:= P(y_{ip} = 0 \mid \eta _p, \boldsymbol{\phi }_i) = 1 - P_i(\eta _p) \end{aligned}$$

The linear predictor *z* in Eq. 1 is expressed in the slope-intercept form rather than the discrimination-difficulty form that one might be more familiar with. The two forms are related. The slope $a_i$ is the discrimination parameter, which quantifies how sensitive an item is to slight differences in individuals’ latent scores: the greater the sensitivity, the higher the discrimination value. In unidimensional IRT, the intercept parameter is the negative product of the slope and the difficulty parameters: $c_i = - a_ib_i$. The difficulty parameter $b_i$ locates the point on the latent continuum at which the probability of responding 1 is 0.5, assuming no guessing.

Under the assumptions of *P* independent individuals and local independence, the marginal log-likelihood for *P* response vectors collected in matrix $\boldsymbol{Y}$ is

3 $$\begin{aligned} \ell (\boldsymbol{\phi } \mid \boldsymbol{Y}) = \sum _{p=1}^{P} \log \left\{ \int \left[ \prod _{i=1}^{I} P_i(\eta )^{y_{ip}} \cdot Q_i(\eta )^{1 - y_{ip}}\right] h(\eta ) d\eta \right\} \end{aligned}$$

where $h(\eta )$ is the standard normal density function for variable $\eta$.

### Parameter estimation with the standard EM algorithm

The integration in Eq. 3 is numerically approximated by a weighted sum over *K* quadrature nodes $\boldsymbol{\eta } = [\eta _1, \dots , \eta _K]'$. For Item *i*, the E-step computes the expected complete data log-likelihood with respect to the posterior distribution $g(\eta _k\mid \boldsymbol{y}_{p}, \boldsymbol{\phi }^{(t)})$ at Iteration $(t+1)$:

4 $$\begin{aligned} Q(\boldsymbol{\phi }_i \mid \boldsymbol{\phi }_i^{(t)})&= \sum _{k=1}^{K} \sum _{p=1}^{P} \Bigl \{ g(\eta _k\mid \boldsymbol{y}_p, \boldsymbol{\phi }^{(t)})\nonumber \\ &\times \left[ y_{ip} \log P_i(\eta _k) + (1 - y_{ip}) \log Q_i(\eta _k)\right] \Bigr \} \end{aligned}$$

The M-step updates iterates $\boldsymbol{\phi }_i^{(t+1)}$ by maximizing or improving Eq. 4; the latter is a more relaxed M-step used in the GEM algorithm.

Furthermore, the summation over *p* in Eq. 4 can be condensed into one summation over the 1 responses and another over the 0 responses:

5 $$\begin{aligned} \tilde{r}_{1ik}^{(t+1)}&= \sum _{p=1}^{P} g(\eta _k\mid \boldsymbol{y}_{p}, \boldsymbol{\phi }^{(t)}) y_{ip} \end{aligned}$$

6 $$\begin{aligned} \tilde{r}_{0ik}^{(t+1)}&= \sum _{p=1}^{P} g(\eta _k\mid \boldsymbol{y}_{p}, \boldsymbol{\phi }^{(t)}) (1 - y_{ip}) \end{aligned}$$

where $\tilde{r}_{1ik}$ and $\tilde{r}_{0ik}$ denote the expected counts of 1 and 0 responses at quadrature node $\eta _k$ for Item *i*.

In practice, the item response matrix for *P* individuals may be compactly represented by *R* unique response patterns and their associated frequencies. Let $\boldsymbol{u}_r = [u_{1r}, u_{2r}, \dots , u_{Ir}]'$ denote the *r*-th pattern and $f_r$ denote the frequency of $\boldsymbol{u}_r$ in the original item response data. Under this pattern-frequency representation, the E-step at Iteration $(t+1)$ computes the expected counts as

7 $$\begin{aligned} \tilde{r}_{1ik}^{(t+1)}&= \sum _{r=1}^{R} f_r \cdot g(\eta _k\mid \boldsymbol{u}_{r}, \boldsymbol{\phi }^{(t)}) u_{ir} \end{aligned}$$

8 $$\begin{aligned} \tilde{r}_{0ik}^{(t+1)}&= \sum _{r=1}^{R} f_r \cdot g(\eta _k\mid \boldsymbol{u}_{r}, \boldsymbol{\phi }^{(t)}) (1 - u_{ir}) \end{aligned}$$

The M-step then finds $\boldsymbol{\phi }_i^{(t+1)}$ that maximizes or improves the Q-function:

9 $$\begin{aligned} Q(\boldsymbol{\phi }_i \mid \boldsymbol{\phi }_i^{(t)}) = \sum _{k=1}^{K} \Bigl \{\tilde{r}_{1ik}^{(t+1)} \log P_i(\eta _k) + \tilde{r}_{0ik}^{(t+1)} \log Q_i(\eta _k) \Bigr \} \end{aligned}$$

The estimation process alternates between the E-step and M-step until a specified convergence criterion is met. One example is to monitor the maximum change in parameter iterates and declare convergence when the difference $|\phi _i^{(t+1)} - \phi _i^{(t)}|$ falls below a small threshold (e.g., $10^{-6}$) for all items.

### Features that motivate the proposed modifications

#### Assumption of conditional independence

The observed responses are conditionally independent given the latent variable. Unless otherwise specified by design, responses from different individuals are also assumed to be independent. These two types of independence allow the complete-data log-likelihood to be expressed as a sum over independent person-level (or item-level) contributions. This structure motivates the use of partial E-steps, where expectations are computed over randomly sampled data subsets (without replacement). This approach reduces the per-iteration computational cost without requiring significant changes to the EM estimation logic.

#### Shape of the log-likelihood surface near the MLE

The log-likelihood surface near the MLE tends to be smooth and locally quadratic. When using Newton-style updates with a small fixed step size, noise introduced by partial E-steps causes the parameter iterates to fluctuate around the MLE within a bounded neighborhood. This characteristic behavior, visualized as a point cloud or noise ball, has been documented in the stochastic optimization literature (e.g., Figure 2 in Gower et al., 2020). Averaging is a common heuristic to reduce this variability and improve estimate precision (Polyak & Juditsky, 1992). A key consideration is whether the iterates have exited the initial phase and entered the noise ball region, where averaging becomes effective. In Section “Stage 1: moving iterates toward the MLE”, we describe how to adaptively detect this transition and present components of the modified EM algorithm.

## Modified EM algorithm

In the standard formulation, maximizing Eq. 9 involves maximizing some form of expectation over a data-defined distribution. Many properties of the log-likelihood function, such as the gradients, are also sums. When applied to large datasets, these operations become computationally expensive because the algorithm must iterate over all observations before performing a single parameter update.

A more efficient approach is to sample smaller subsets of the data and perform the same operations on each subset, yielding noisier yet "good enough" estimates. This motivates a partial E-step strategy, where the E-step is performed on data subsets to compute subset-level expected counts. Moreover, the M-step can be relaxed as in GEM, where parameters are updated incrementally using partial information—such as gradients or Hessians based on subset-level expectations—rather than solving for an exact M-step solution. These two modifications—(a) performing partial E-steps and (b) updating parameters incrementally in the M-step—form the basis of the modified algorithm.

### Overview

**Fig. 1:**
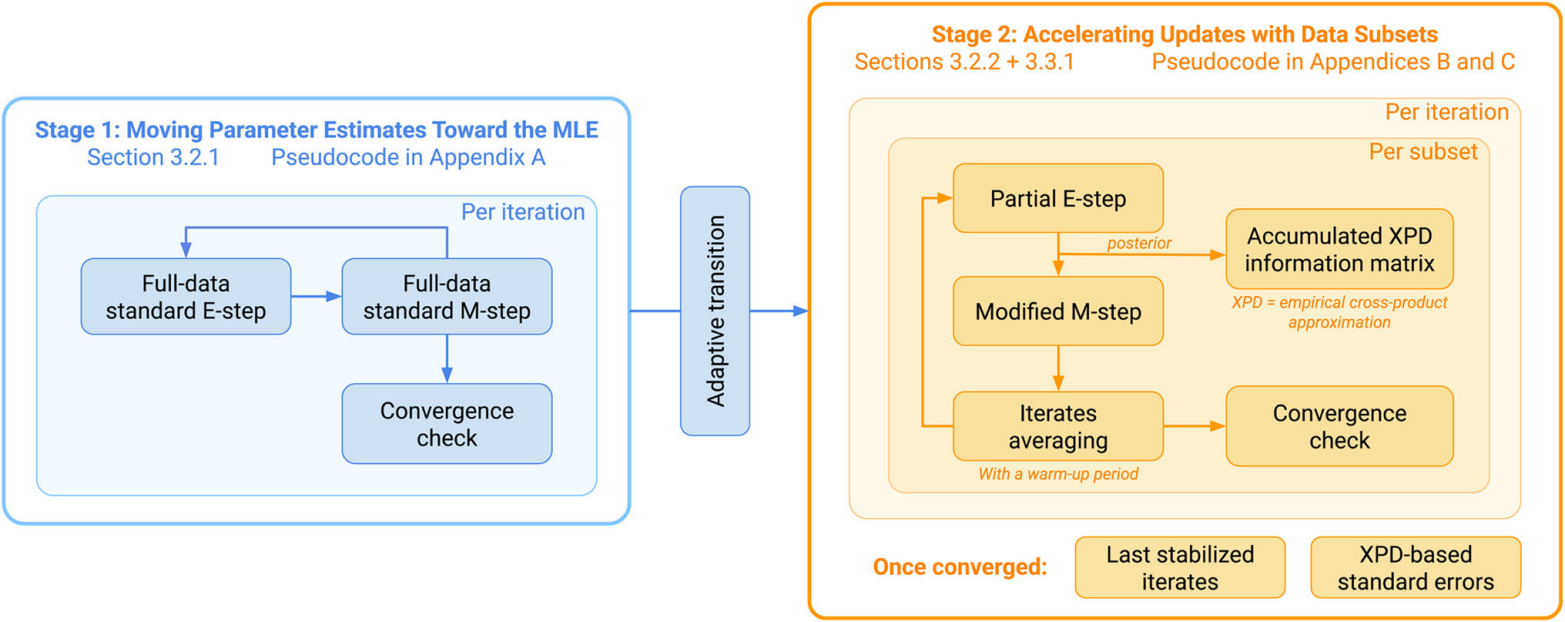
Main components of the modified EM algorithm

The proposed algorithm uses two stages to obtain parameter estimates (Fig. 1). In Stage 1, it uses the standard EM algorithm to move the iterates toward the neighborhood of the MLE. Once a pre-specified stability criterion is met, it transitions to Stage 2 to operate on data subsets to accelerate convergence. Also during Stage 2, it incrementally constructs a cross-product approximation to the observed information matrix, which stabilizes upon convergence and is then used to compute standard errors. We provide pseudocode in the appendices to outline key steps and default hyperparameter settings.

**Fig. 2:**
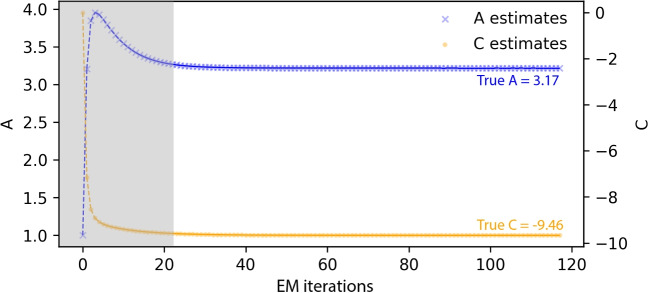
Example EM estimation trajectory

### Parameter estimation: a two-stage approach

#### Stage 1: moving iterates toward the MLE

Recall that the standard EM algorithm is efficient in early iterations, making substantial improvements to parameter estimates. However, as the iterates approach the MLE, the algorithm continues to process the full dataset in each iteration while yielding diminishing improvements, making later updates computationally more expensive.

The proposed algorithm addresses this inefficiency by leveraging the early performance of standard EM and then switching to a more computationally efficient approach. To determine when to switch from full-data standard EM (Stage 1) to the subset-based modified EM (Stage 2), the algorithm uses a log-likelihood-based switching rule. This rule detects when the iterates have "gotten over the hump"—that is, when they have moved past the initial large updates and entered the neighborhood of the MLE. Figure 2 shows an EM estimation trajectory for an item with a high slope (3.17) and extreme difficulty (2.99) and intercept (-9.46) values, with the approximate hump- or elbow-shaped region shaded for illustration.

The adaptive transition ensures that major updates driven by full-data EM have occurred and allows Stage 2 to refine parameter estimates with lower computational and time costs. Appendix A outlines Stage 1’s implementation.

##### Adaptive transition to Stage 2

The transition from Stage 1 to Stage 2 is governed by a switching rule based on the exponentiated negative difference in observed (marginal) log-likelihood values (Tian, Cai, Thissen, & Xin, 2013). The quantity $\delta ^{(j)}$ at Iteration *j* is defined as

10 $$\begin{aligned} \delta ^{(j)} = \exp \left[ -\left( \texttt {LL}^{(j)} - \texttt {LL}^{(j-1)}\right) \right] \end{aligned}$$

where $\texttt {LL}^{(j)}$ and $\texttt {LL}^{(j-1)}$ are the log-likelihood values at Iterations *j* and $j-1$. At the first iteration, where $\texttt {LL}^{(j-1)}$ is not yet defined, $\delta ^{(j)}$ is set to 0. The transition occurs when $\delta ^{(j)}$ exceeds a predefined threshold in the interval (0, 1]. A threshold closer to one better ensures that the iterates are within or near the MLE neighborhood before switching, but it may prolong the costly Stage 1 beyond necessity. In comparison, a lower threshold may not guarantee proximity across all parameters but enables an earlier transition that benefits from Stage 2 updates. Our implementation uses 0.90 as a conservative default when the sample size exceeds 10,000. In Section “Simulation 1: estimating item parameters”, with $N = 60,000$, we examine how varying this threshold affects runtime and performance. For larger samples, the threshold can be much lower without compromising performance (see Section “Results”).

##### Early stopping condition

When only Stage 1 is run, convergence is assessed based on whether the largest absolute changes in the A and C iterates fall below a predefined threshold (e.g., $10^{-6}$). If this condition is met, the algorithm exits Stage 1, and the last iterates are taken as the final parameter estimates. If Stage 2 is enabled, and the condition is not met, the algorithm transitions to Stage 2.

#### Stage 2: accelerating updates with data subsets

Stage 2 accelerates convergence by updating parameters using randomly partitioned data subsets. For each subset, it performs a partial E-step followed by a modified M-step. Appendix B outlines Stage 2’s implementation.

##### Creating data subsets

The subsets are created following shuffling and partitioning. In the shuffling step, the row indices of the full item response data are shuffled to mimic random selection and minimize data-ordering effects (Goodfellow, Bengio, & Courville, 2016). Faster convergence has also been observed if the data subsets were randomly ordered for each iteration (Bengio, 2012). In the partitioning step, the vector of shuffled indices is partitioned into *M* subsets. For example, 5% of a sample size of 10,000 translates to a subset size of 500 and a *M* of 20. In the case where the sample size is not divisible by the subset size, the remaining data can be its own subset. Each subset is then summarized into a response pattern matrix $\boldsymbol{U}$ and frequency vector $\boldsymbol{f}$.

##### Partial E-steps

The partial E-step follows the same procedure as in the standard EM algorithm. The difference is that each E-step is performed on a Subset *s*, consisting of response pattern data $\boldsymbol{U}^{(s)}$ from $P^{(s)}$ individuals randomly drawn from the full set of *P* individuals. Each partial E-step yields subset-level expected counts of 1 and 0 responses for *I* items across *K* quadrature nodes:

11 $$\begin{aligned} \tilde{r}_{1ik}^{(t+1,s)}&= \sum _{r} f_r^{(s)} \cdot g\left( \eta _k \mid \boldsymbol{u}_r^{(s)}, \boldsymbol{\phi }^{(t)}\right) \cdot u_{ir}^{(s)} \end{aligned}$$

12 $$\begin{aligned} \tilde{r}_{0ik}^{(t+1,s)}&= \sum _{r} f_r^{(s)} \cdot g\left( \eta _k \mid \boldsymbol{u}_r^{(s)}, \boldsymbol{\phi }^{(t)}\right) \cdot \left( 1 - u_{ir}^{(s)}\right) \end{aligned}$$

where $f_r^{(s)}$ denotes the frequency of the *r*-th pattern in Subset *s*.

##### Modified M-steps

Each partial E-step is followed by a modified M-step that performs a Newton-style update for Item *i*’s parameters:

13 $$\begin{aligned} \boldsymbol{\phi }^{(t+1)}_i = \boldsymbol{\phi }^{(t)}_i + \gamma \left( \boldsymbol{H}^{(t+1)}_i \right) ^{-1}\boldsymbol{\nabla }^{(t+1)}_i \end{aligned}$$

In Eq. 13, $\boldsymbol{\nabla }^{(t)}_{i}$ and $\boldsymbol{H}^{(t)}_{i}$ denote the gradient and Hessian of the expected complete data log-likelihood.^1^ The fixed step size $\gamma \in (0, 1]$ controls the magnitude of the update ($\gamma = 0.5$ by default).

##### Gradients of expected complete-data log-likelihood

For Item *i*, the gradients of Eq. 9 with respect to the item parameters are

14 $$\begin{aligned} \frac{\partial Q}{\partial a_i}&= \sum _k \left[ \tilde{r}_{1ik} - \tilde{n}_{ik} P_i(\eta _k) \right] \eta _k \end{aligned}$$

15 $$\begin{aligned} \frac{\partial Q}{\partial c_i}&= \sum _k \left[ \tilde{r}_{1ik} - \tilde{n}_{ik} P_i(\eta _k) \right] \end{aligned}$$

Here, we omit the superscripts to simplify notations. $P_i(\eta _k)$ denotes the response function (Eq. 1) evaluated at $\eta _k$, and $\tilde{n}_{ik} = \tilde{r}_{1ik} + \tilde{r}_{0ik}$ is the expected number of individuals at $\eta _k$. The quantities $\tilde{r}_{1ik}$ and $\tilde{r}_{0ik}$ are the expected counts of 1 and 0 responses obtained from the partial E-step.

##### Hessian of expected complete-data log-likelihood

Elements of the Hessian matrix are given by

16 $$\begin{aligned} \frac{\partial ^2 Q}{\partial a_i^2}&= - \sum _k \tilde{n}_{ik} P_i(\eta _k) Q_i(\eta _k) \eta _k^2 \end{aligned}$$

17 $$\begin{aligned} \frac{\partial ^2 Q}{\partial c_i^2}&= - \sum _k \tilde{n}_{ik} P_i(\eta _k) Q_i(\eta _k) \end{aligned}$$

18 $$\begin{aligned} \frac{\partial ^2 Q}{\partial a_i \partial c_i}&= - \sum _k \tilde{n}_{ik} P_i(\eta _k) Q_i(\eta _k) \eta _k \end{aligned}$$

where $Q_i(\eta _k) = 1 - P_i(\eta _k)$, as defined in Eq. 2.

##### Iterates averaging

With unidimensional 2PL IRT models, the log-likelihood surface near the MLE tends to be approximately quadratic. Within this region, M-step updates with a fixed step size often oscillate around the MLE. Instead of tuning the step size, we approximate the MLE by computing a running average of iterates to track the center of the oscillation and monitor convergence. The averaged values are fed back into the E-step at each subsequent iteration.

Averaging starts after a fixed warm-up period (e.g., 30 updates), by which point the iterates are more likely to be oscillating around the MLE rather than still trending toward it. Depending on the choice of the transition threshold, some trending toward the MLE may still occur in early Stage 2 iterations, particularly for parameters with extreme values. The warm-up period serves as a buffer to reduce the risk of premature averaging.

Once the averaged values stabilize, the most recent values are retained as the final estimates. Stability is determined by whether the variance of recent averaged values falls below a threshold (e.g., $10^{-6}$). Appendix C outlines the core functions used for averaging and stability checking.

##### Early stopping condition

In Stage 2, convergence is assessed by checking whether the largest absolute changes in the averaged A and C iterates fall below a predefined tolerance (e.g., $10^{-6}$). If this condition is met, the algorithm exits Stage 2 and returns the final parameter estimates.

### Standard error estimation

The EM framework does not produce the parameter information matrix or error covariance matrix, and hence provides no standard errors (SEs) upon convergence.

#### Computing SEs based on the empirical cross-product information matrix

One solution is the empirical cross-product (XPD) approximation (Meilijson, 1989), which computes the information matrix as the outer product of the score function of the marginal log-likelihood. Compared to alternative methods, XPD is chosen for the following reasons. The expected information matrix, while useful as a gold-standard reference for simulation studies, becomes computationally prohibitive in practice as the number of items grows. For example, with 30 items, its computational complexity scales with the $2^{30}$ possible response patterns, whereas the XPD approach remains tractable (Maydeu-Olivares, 2013; Monroe, 2019). Compared to the observed information matrix and the supplemented EM approach, the building blocks of the XPD information matrix can be accumulated over subsets by exploiting numerical byproducts of the partial E-step (e.g., $g_{kr}$ in Eqs. 19 and 20) and the additivity of information over independent observations.

In Stage 2, the modified algorithm computes the XPD matrix incrementally by accumulating score outer-products over disjoint data subsets. Upon convergence, the inverse of the final XPD matrix yields the parameter error covariance matrix. SEs are then obtained as the square roots of the diagonal elements of the covariance matrix. The subsections below present key numeric results. Appendix D outlines the implementation for accumulating the XPD information matrix and computing standard errors.

##### Gradients of marginal log-likelihood

The gradients with respect to Item *i*’s parameters are:

19 $$\begin{aligned} \frac{\partial \ell _r}{\partial a_i}&= \sum _{k=1}^K g_{kr} \left[ u_{ir} - P_i(\eta _k)\right] \eta _k \end{aligned}$$

20 $$\begin{aligned} \frac{\partial \ell _r}{\partial c_i}&= \sum _{k=1}^K g_{kr} \left[ u_{ir} - P_i(\eta _k)\right] \end{aligned}$$

where $g_{kr}$ denotes the posterior probability $g(\eta _k \mid \boldsymbol{u}_r, \boldsymbol{\phi })$.

##### XPD information matrix

Let the sample gradient vector for Pattern *r* be

21 $$\begin{aligned} \boldsymbol{v}_r = \left[ \frac{\partial \ell _r}{\partial a_1},\ \frac{\partial \ell _r}{\partial c_1},\ \dots ,\ \frac{\partial \ell _r}{\partial a_I},\ \frac{\partial \ell _r}{\partial c_I} \right] ' \end{aligned}$$

The XPD information matrix is then approximated by

22 $$\begin{aligned} \text {I}_{\text {xpd}}(\boldsymbol{\phi }) = \sum _{r=1}^R f_r \left[ \boldsymbol{v}_r\left( \boldsymbol{v}_r\right) ' \right] \end{aligned}$$

#### Computing SEs based on the fisher expected information matrix

To evaluate the accuracy and precision of XPD-based SE estimates in later simulation studies, an additional set of SEs is computed from the Fisher expected information (FIS) matrix. These serve as a theoretical benchmark or gold standard for comparison.

##### Fisher expected information matrix

The FIS is defined as

23 $$\begin{aligned} \mathcal {F}(\boldsymbol{\phi }) = \mathcal {J}(\boldsymbol{\phi })' \left\{ \textrm{diag}[\boldsymbol{\pi }(\boldsymbol{\phi })] \right\} ^{-1} \mathcal {J}(\boldsymbol{\phi }) \end{aligned}$$

In Eq. 23, $\boldsymbol{\phi }$ is a vector collecting all item parameters. The matrix $\mathcal {J}(\boldsymbol{\phi })$ contains the gradients of the marginal log-likelihood, where each row corresponds to a response pattern and each column to a model parameter. Each non-zero entry in the diagonal matrix $\textrm{diag}[\boldsymbol{\pi }(\boldsymbol{\phi })]$ shows the probability of observing a given pattern under the 2PL IRT model. The number of unique response patterns increases exponentially with the number of items, following $2^{I}$ where *I* is the number of items. For example, 12 binary items yield $2^{12} = 4096$ possible patterns.

In simulation studies, FIS can be evaluated at the true parameter values, denoted by $\mathcal {F}(\boldsymbol{\phi }_0)$. Given a sample size of *N*, the matrix $[N\mathcal {F}(\boldsymbol{\phi }_0)]^{-1}$ is the parameter error covariance matrix. The square roots of its diagonal elements provide the gold-standard SEs (Cai, 2008; Tian et al., 2013). Appendix E provides a R function for computing FIS-based SEs using output from flexMIRT 3.72 (Cai, 2024).

## Simulation studies

We used the first two simulation studies to evaluate time to convergence, parameter recovery, and standard error estimation performance of the proposed algorithm relative to standard EM. We designed a third study to mimic a large-scale testing scenario, in which 1 million examinees were randomly assigned to 40-item forms drawn from a pool of 100 items. We used the fourth study as a robustness check on performance under less ideal data conditions.

The algorithm implementation was done in Python 3.13.2^2^, and the function in Appendix E was written in R 4.3.1. All simulations were run on an M3 Mac with 16 GB RAM. Reported times reflect performance under this configuration and may vary depending on specific hardware and software environments.

### Simulation 1: estimating item parameters

In Study 1, we evaluated the extent to which the proposed algorithm reduced time to convergence (measured as runtime) and recovering item parameters under different data conditions.

#### Design

Study 1 varied three factors: (a) percentage subset size (10%, 20%, 30%, 50%, or 100% of the sample size), (b) number of items (18, 36, or 54), and (c) transition thresholds (0.10, 0.50, or 0.90). In total, there were $4 \times 3 \times 3 + 3 = 39$ conditions. The last three conditions corresponded to standard EM runs using the full dataset, where only the number of items varied. Hyperparameters not manipulated in the design were held at their default values, as noted in Appendices A to  C.

Each condition was replicated 100 times; all replications were run until convergence. For each replication, binary responses were simulated for 60,000 individuals under a unidimensional 2PL IRT model, given true item parameters and a standard normal distribution for the latent variable. The next section describes how the true item parameters were generated.

#### Grid sampling of true item parameters

The true A (slope) and B (difficulty) parameter values were first randomly sampled at three levels: low, medium, and high. The levels of A were selected based on literature suggesting that a reasonable range for slope values is between 0.8 and 2.5 (Baker & Kim, 2004; de Ayala, 2009). The levels of B were selected considering that approximately 99.73% of values in a standard normal distribution fall within $\pm 3$ standard deviations. Table 1 shows the sampling ranges for these levels.

These levels formed a 3-by-3 sampling grid. Within each cell, an equal number of item parameters was sampled from a uniform distribution over the specified range. For the 18-item condition, two items were sampled from the Low A and Low B cell, and the same procedure was repeated for the remaining eight cells. For the 36-item condition, six items were sampled per cell.

**Table 1:** Study 1: Level ranges for item parameters

Item parameter	Low	Medium	High
A (discrimination/slope)	[0.50, 1.50)	[1.50, 2.50)	[2.50, 3.50]
B (difficulty)	[-3.00, -1.50)	[-1.50, 1.50)	[1.50, 3.00]
C (intercept)	[-10.50, -3.75)	[-3.75, 3.75)	[3.75, 10.50]

**Table 2:** Study 1: True item parameters for 18-item conditions

	True value				Level		
Item	A	B	C		A	B	C
1	0.64	$-1.67$	1.06		low	low	med
2	1.43	$-2.33$	3.34		low	low	med
3	0.89	$-0.73$	0.65		low	med	med
4	1.16	$-0.02$	0.03		low	med	med
5	1.46	2.70	$-3.96$		low	high	low
6	0.96	2.70	$-2.58$		low	high	med
7	1.54	$-1.85$	2.85		med	low	med
8	1.50	$-2.56$	3.85		med	low	high
9	2.11	1.24	$-2.62$		med	med	med
10	1.80	$-0.75$	1.36		med	med	med
11	2.17	2.98	$-6.46$		med	high	low
12	1.97	1.68	$-3.32$		med	high	med
13	3.42	$-1.58$	5.40		high	low	high
14	2.78	$-2.22$	6.17		high	low	high
15	2.65	$-1.46$	3.87		high	med	high
16	2.82	1.47	$-4.16$		high	med	low
17	3.01	2.81	$-8.48$		high	high	low
18	2.57	1.93	$-4.95$		high	high	low

*Note.* $C = -A \times B$. Parameter levels are based on cutoffs in Table 1

**Table 3:** Study 1: True item parameters for 36-item conditions

	True value					True value		
Item	A	B	C		Item	A	B	C
1	0.64	$-1.67$	1.06		19	1.97	0.79	$-1.55$
2	1.43	$-2.33$	3.34		20	2.42	$-0.32$	0.78
3	0.89	$-2.61$	2.32		21	2.43	2.25	$-5.46$
4	1.16	$-2.26$	2.62		22	2.30	2.84	$-6.55$
5	1.46	0.90	$-1.32$		23	1.98	2.77	$-5.49$
6	0.96	0.90	$-0.86$		24	1.78	2.01	$-3.57$
7	0.54	0.81	$-0.44$		25	2.61	$-2.22$	5.77
8	0.50	$-0.62$	0.31		26	3.15	$-1.65$	5.20
9	1.11	2.87	$-3.19$		27	2.60	$-2.53$	6.59
10	0.80	1.87	$-1.50$		28	2.58	$-1.59$	4.11
11	1.17	2.98	$-3.48$		29	3.46	$-0.93$	3.20
12	0.97	1.68	$-1.63$		30	3.45	$-0.17$	0.58
13	2.42	$-1.58$	3.82		31	2.76	0.51	$-1.41$
14	1.78	$-2.22$	3.95		32	2.88	1.46	$-4.19$
15	1.65	$-2.98$	4.93		33	3.17	2.99	$-9.46$
16	1.82	$-1.51$	2.76		34	3.47	1.77	$-6.16$
17	2.01	1.13	$-2.27$		35	2.94	2.51	$-7.38$
18	1.57	$-0.65$	1.01		36	3.15	2.07	$-6.54$

*Note.* $C = -A \times B$. Parameter levels are omitted

**Table 4:** Study 1: True item parameters for 54-item conditions

	True value					True value					True value		
Item	A	B	C		Item	A	B	C		Item	A	B	C
1	0.64	$-1.67$	1.06		19	1.97	$-1.86$	3.66		37	3.17	$-1.92$	6.09
2	1.43	$-2.33$	3.34		20	2.42	$-2.41$	5.84		38	3.28	$-2.52$	8.27
3	0.89	$-2.61$	2.32		21	2.43	$-2.25$	5.47		39	2.60	$-1.75$	4.54
4	1.16	$-2.26$	2.62		22	2.30	$-1.66$	3.81		40	2.53	$-2.64$	6.67
5	1.46	$-1.80$	2.63		23	1.98	$-1.73$	3.43		41	3.36	$-2.92$	9.82
6	0.96	$-1.80$	1.72		24	1.78	$-2.49$	4.42		42	2.69	$-2.29$	6.16
7	0.54	0.81	$-0.44$		25	1.61	0.07	$-0.11$		43	2.74	$-0.42$	1.16
8	0.50	$-0.62$	0.31		26	2.15	1.20	$-2.58$		44	2.65	$-1.09$	2.89
9	1.11	1.24	$-1.38$		27	1.60	$-0.56$	0.91		45	2.60	$-1.04$	2.71
10	0.80	$-0.75$	0.60		28	1.58	1.31	$-2.07$		46	2.64	$-1.41$	3.73
11	1.17	1.46	$-1.71$		29	2.46	$-0.93$	2.28		47	3.23	0.53	$-1.71$
12	0.97	$-1.13$	1.09		30	2.45	$-0.17$	0.41		48	2.94	$-1.13$	3.31
13	1.42	2.92	$-4.13$		31	1.76	2.50	$-4.42$		49	2.68	2.41	$-6.46$
14	0.78	2.28	$-1.77$		32	1.88	2.98	$-5.59$		50	2.77	2.57	$-7.12$
15	0.65	1.52	$-1.00$		33	2.17	2.99	$-6.47$		51	2.87	1.92	$-5.50$
16	0.82	2.99	$-2.46$		34	2.47	1.77	$-4.38$		52	3.38	1.75	$-5.92$
17	1.01	2.81	$-2.85$		35	1.94	2.51	$-4.87$		53	2.60	2.96	$-7.70$
18	0.57	1.93	$-1.09$		36	2.15	2.07	$-4.47$		54	3.46	1.69	$-5.83$

*Note.* $C = -A \times B$. Parameter levels are omitted

Next, the true A and B values were used to compute the true C (intercept) values using the formula $C = -A \times B$. The full range of possible C values, $[-10.50,10.50]$, was derived from the outer bounds of $A \in [0.50,3.50]$ and $B \in [-3.00,3.00]$. The cutoffs at $\pm 3.75$ divided this range into three approximately equal-width intervals for later subgroup summaries of evaluation metrics. As a reminder, the A and C parameters were those being estimated. Tables 2, 3 and 4 present the true A, B, and C parameters used in the 18-item, 36-item, and 54-item conditions.

#### Evaluation metrics

We evaluated the proposed algorithm based on its reduction in time to convergence and parameter recovery performance. The latter included bias and root-mean-square error (RMSE).

**Fig. 3:**
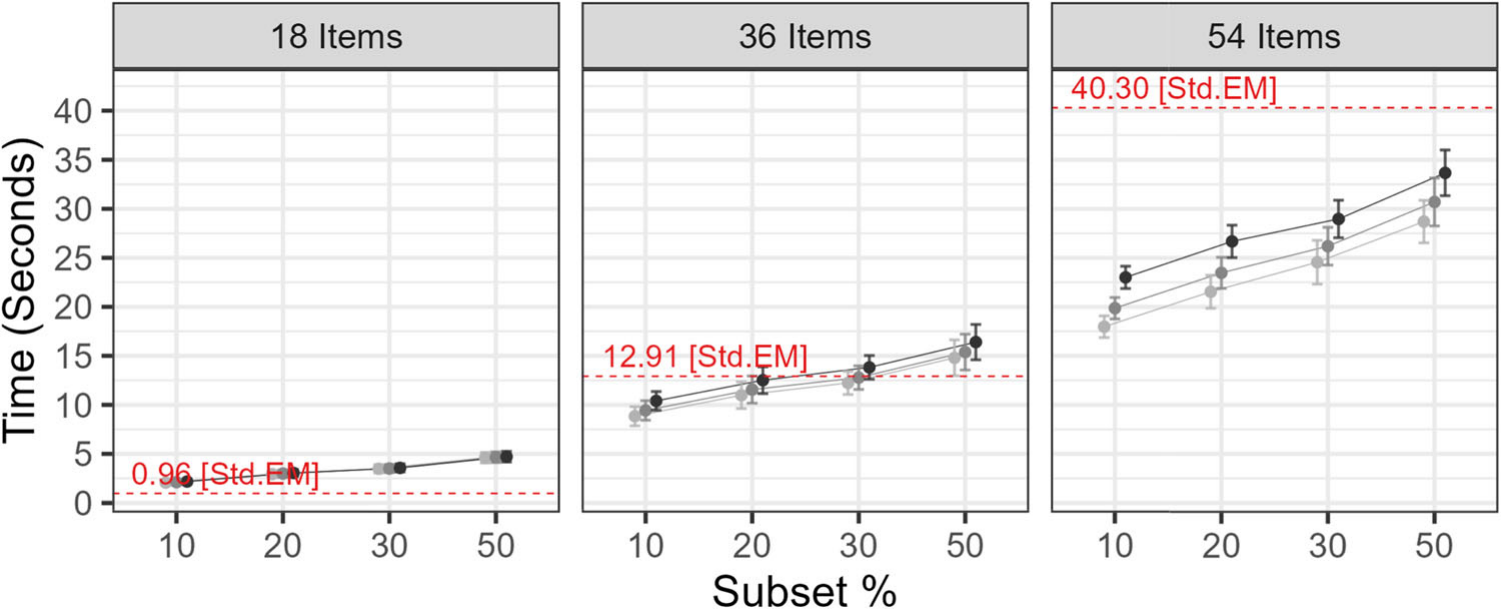
Mean and SD of time to convergence by condition. *Note.* The 0.99 threshold is included as a highly conservative reference and is not included in Simulation 1

##### Time to convergence

24 $$\begin{aligned} \hspace{0.83328pt}\overline{\hspace{-0.83328pt}\text {T}\hspace{-0.83328pt}}\hspace{0.83328pt}_m&= \frac{1}{R}\sum _{r=1}^{R} \text {Time}_r \end{aligned}$$

25 $$\begin{aligned} \text {\% Time Change}&= \left( \hspace{0.83328pt}\overline{\hspace{-0.83328pt}\text {T}\hspace{-0.83328pt}}\hspace{0.83328pt}_m - \hspace{0.83328pt}\overline{\hspace{-0.83328pt}\text {T}\hspace{-0.83328pt}}\hspace{0.83328pt}_{f}\right) / \, \hspace{0.83328pt}\overline{\hspace{-0.83328pt}\text {T}\hspace{-0.83328pt}}\hspace{0.83328pt}_{f} \times 100\% \end{aligned}$$

where $\hspace{0.83328pt}\overline{\hspace{-0.83328pt}\text {T}\hspace{-0.83328pt}}\hspace{0.83328pt}_{f}$ is the mean runtime based on the full-data standard EM, and $\hspace{0.83328pt}\overline{\hspace{-0.83328pt}\text {T}\hspace{-0.83328pt}}\hspace{0.83328pt}_{m}$ is the mean runtime based on the proposed algorithm. All times are in seconds. *r* denotes the *r*th replication, and *R* is the total number of replications.

##### Parameter recovery

26 $$\begin{aligned} \text {Bias}\left( \hat{\phi }\right)&= \frac{1}{R}\sum _{r=1}^{R}\left( {\hat{\phi }}_r-\phi \right) \end{aligned}$$

27 $$\begin{aligned} \text {RMSE}\left( \hat{\phi }\right)&= \sqrt{\frac{1}{R}\sum _{r=1}^{R}\left( \hat{\phi }_r-\phi \right) ^2} \end{aligned}$$

where $\phi$ is the true parameter generated following Section “Grid sampling of true item parameters”, and ${\hat{\phi }}_r$ is the parameter estimate from the *r*th replication.

#### Results

##### Reduction in time to convergence

Figure 3 summarizes the effects of varying transition thresholds on time across simulation conditions. We also include the highly conservative threshold of 0.99 as a stress-test case, which delays Stage 2 with negligible differences in recovery performance. For all item counts, larger thresholds led to greater runtime.

Table 5 summarizes time-related evaluation metrics for the 18-item and 54-item conditions, which are the lowest and highest item counts evaluated in Study 1.^3^ The proposed algorithm reduced runtime in the 36- and 54-item conditions, with results from the 54-item condition showing consistent time savings across conditions. These results suggest reductions scaled with item count, and the proposed algorithm is more efficient when applied to a larger number of items. This may be because the cost of summing over response patterns increases with item count. With more items, individuals are more likely to produce unique or near-unique response patterns, resulting in a much larger pattern matrix. In such cases, a subset-based approach reduces the per-iteration computational cost and is particularly advantageous when the response pattern matrix begins to resemble the full item response matrix.

**Table 5:** Study 1: Mean and SD of time to convergence in seconds

		18 Items					54 Items			
Threshold		Subset %	*M*	*SD*	Change %		Subset %	*M*	*SD*	Change %
0.10		10	2.07	0.28	+ 114.71		10	17.97	1.11	- 55.41
		20	2.94	0.37	+ 204.71		20	21.54	1.69	- 46.56
		30	3.48	0.42	+ 260.91		30	24.55	2.24	- 39.08
		50	4.61	0.54	+ 378.21		50	28.70	2.17	- 28.79
0.50		10	2.11	0.28	+ 119.05		10	19.86	1.09	- 50.71
		20	2.99	0.37	+ 210.20		20	23.47	1.58	- 41.76
		30	3.51	0.42	+ 263.94		30	26.19	1.92	- 35.02
		50	4.66	0.54	+ 383.35		50	30.70	2.44	- 23.82
0.90		10	2.18	0.28	+ 125.79		10	23.01	1.14	- 42.91
		20	3.04	0.37	+ 216.05		20	26.68	1.65	- 33.80
		30	3.56	0.42	+ 269.98		30	28.96	1.91	- 28.13
		50	4.71	0.54	+ 389.27		50	33.67	2.33	- 16.47

*Note.* Change % is calculated as $(M - M_{std.em})/M_{std.em} \times 100\%$

##### Bias and RMSE for estimated item parameters

We focus on results from the 54-item condition, where time reductions occurred consistently across subset sizes. Patterns in the 18-item and 36-item conditions were similar to those in the 54-item condition.

**Fig. 4:**
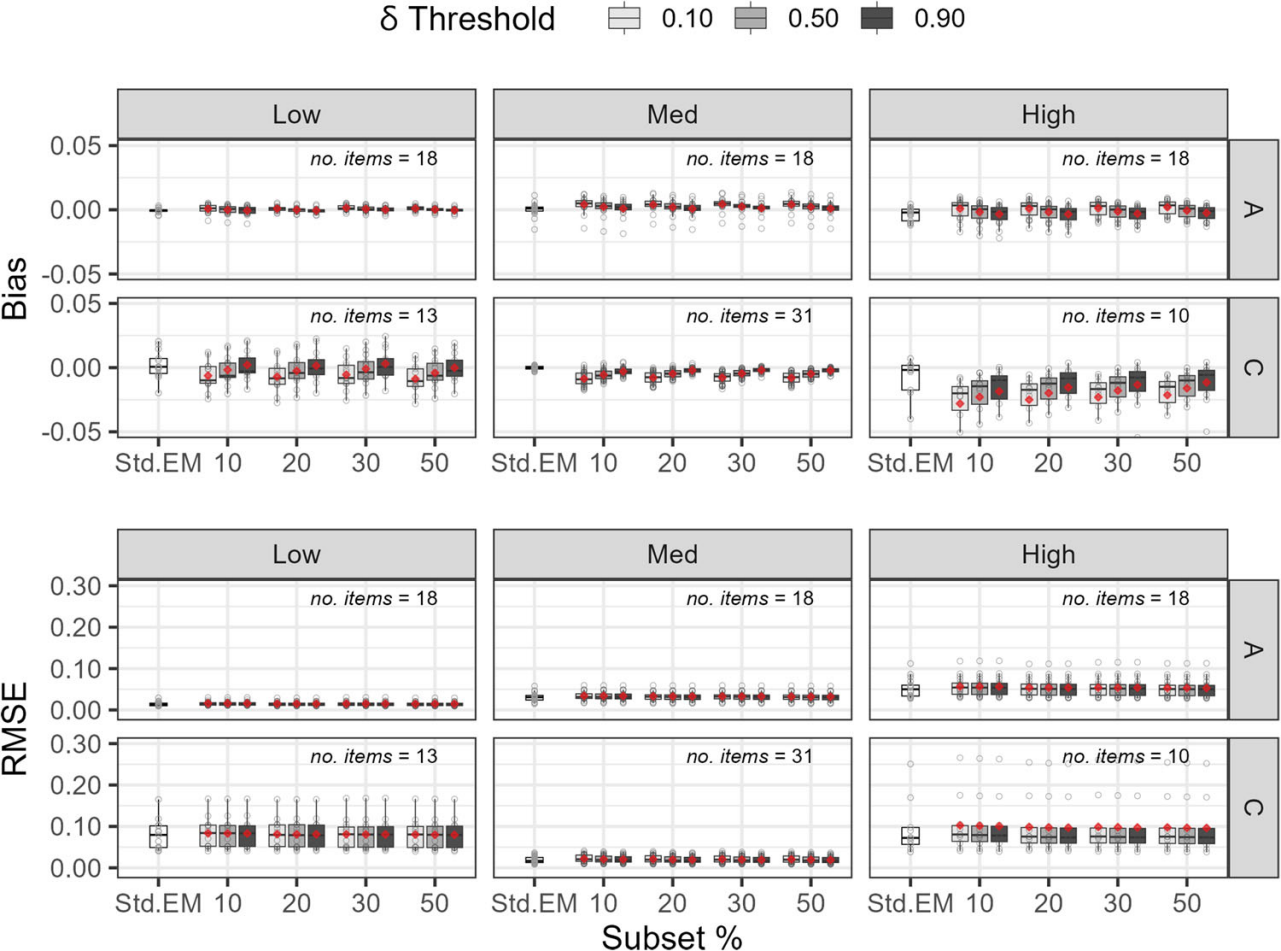
Study 1 (54 items): Bias and RMSE for parameter estimates. *Note.* *Grey circles*: individual bias or RMSE values; *red diamonds*: mean value. The number of items categorized as low, medium (med), or high on the A or C parameter is noted in the upper right of each panel

Figure 4 presents bias and RMSE values, broken down by subset size, parameter type, and parameter magnitude levels defined in Section “Grid sampling of true item parameters”. In the figure, grey circles represent individual values, red diamonds show the mean for each condition, and the number of items whose A or C parameters were categorized as low, medium (med), or high is noted in each panel.

We make several observations from this figure. First, for subset sizes of 20% and above, bias and RMSE values were closer to those of standard EM. Second, the true slope parameters (A) were easier to recover than the intercept parameters (C), as indicated by the lower and less dispersed bias and RMSE. Third, parameters with extreme values were more challenging to estimate compared to parameters with moderate values. This was particularly evident when both the slope (A) and difficulty (B) parameters were high, resulting in extreme and hard-to-estimate intercept parameters (C). Estimation in these cases was challenging because the items were highly sensitive to differences in the latent score, but only for individuals at the extremes of the distribution, where few are located under a standard normal distribution.

Supplemental Tables S1 to S5 report mean estimate, standard deviation, bias, and RMSE by individual parameter under the 54-item condition with the 0.90 threshold. All values were computed using unrounded data but are reported to two decimal places for readability. The patterns observed in Fig. 4, as well as those under the 18- and 36-item conditions, are similarly reflected here. For example, larger bias and RMSE values tended to occur for parameters with extreme true values, particularly among the C parameters.

### Simulation 2: Estimating standard errors

In Study 2, we evaluated the performance of SE estimation under the proposed algorithm. The methods for computing SEs–based on the Fisher expected information matrix (FIS) and the empirical cross-product approximation (XPD)—are described in Section “Standard error estimation”.

**Table 6:** Study 2: True item parameters and standard errors

Item	A	*SE*(a)	C	*SE*(c)
1	0.68	0.015	1.60	0.013
2	1.27	0.022	2.24	0.021
3	1.56	0.026	2.39	0.024
4	1.22	0.019	1.57	0.016
5	1.38	0.020	1.14	0.015
6	1.80	0.024	0.21	0.014
7	1.33	0.018	$-0.16$	0.012
8	2.02	0.029	$-1.19$	0.018
9	1.16	0.018	$-1.23$	0.014
10	1.11	0.019	$-1.70$	0.016
11	0.94	0.017	$-1.66$	0.015
12	0.80	0.017	$-1.83$	0.015

*Note.* $B = -C/A$. B values (item difficulties) range between -2.35 and 2.29

**Fig. 5:**
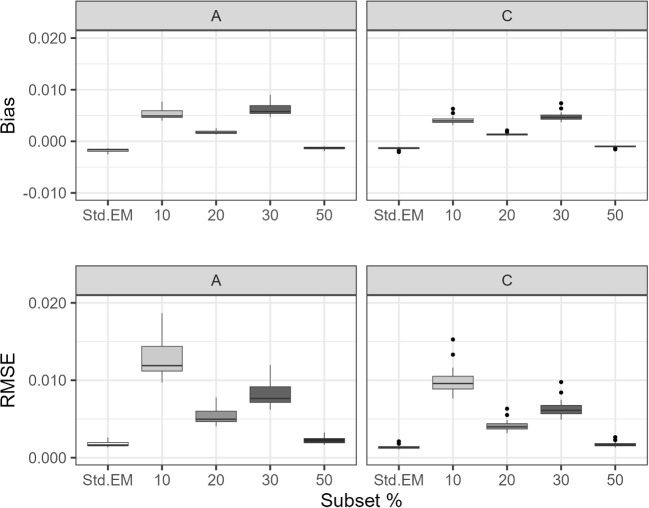
Study 2 (0.90 threshold): Bias and RMSE for XPD-based SE estimates

#### Design

Study 2 varied the percentage subset size (10%, 20%, 30%, 50%, or 100%) while keeping the transition threshold at the 0.90 default. Hyperparameters not varied in the design were held at their default values, as noted in Appendices A to  C.

Each condition was replicated 1000 times; all replications were run until convergence. For each replication, dichotomously scored responses to 12 items were simulated for 60,000 individuals, assuming a unidimensional 2PL IRT model and a standard normal distribution for the latent variable. Table 6 presents the true parameter values and their FIS-based SEs.

#### Evaluation metrics

For each true parameter, we computed the FIS-based standard error $\text {SE}_{\text {fis}}(\phi )$. For each condition, we also computed the mean of XPD-based estimated SEs across replications. The following metrics quantify the difference between $\text {SE}_{\text {fis}}(\phi )$ and the estimated SE:

28 $$\begin{aligned} \text {Bias}\left[ \text {SE}(\phi )\right]&= \frac{1}{R}\sum _{r=1}^{R}{\text {SE}(\hat{\phi }_r)} - \text {SE}_{\text {fis}}(\phi ) \end{aligned}$$

29 $$\begin{aligned} \text {RMSE}\left[ \text {SE}(\phi )\right]&= \sqrt{\frac{1}{R}\sum _{r=1}^{R}\left[ \text {SE}(\hat{\phi }_r) - \text {SE}_{\text {fis}}(\phi )\right] ^2} \end{aligned}$$

#### Results

We estimated SEs using XPD and compared them to gold-standard SEs derived from FIS. Figure 5 shows the distribution of bias and RMSE values by condition. Under standard EM, both bias and RMSE were near zero. Under the proposed algorithm, upward bias and increased RMSE were observed, particularly for the 10% subset condition. However, for subset sizes of 20% and larger, the absolute magnitudes of both metrics remained close to or below 0.01. Supplemental Tables S6 to S10 report the gold-standard SE, mean estimated SE, bias, and RMSE by parameter. All values were computed using unrounded data but are reported to three decimal places for readability.

### Simulation 3: A large-scale form-based testing scenario

Given the results from Studies 1 and 2, Study 3 served to mimic a realistic large-scale operational setting. In Study 3, we demonstrated the runtime advantage and scalability of the modified EM algorithm by simulating a scenario in which one million respondents were randomly assigned to complete one of ten 40-item forms drawn from a 100-item pool.

#### Design

Two factors were varied: (a) subset size percentage (10%, 20%, 30%, 50%, 100%) and (b) transition threshold (0.1, 0.5, 0.9). A single replication was run until convergence per condition. For each replication, binary responses were generated for 1 million individuals under a unidimensional 2PL IRT model, given true item parameters and a standard normal distribution for the latent variable.

#### Construction of forms

**Fig. 6:**
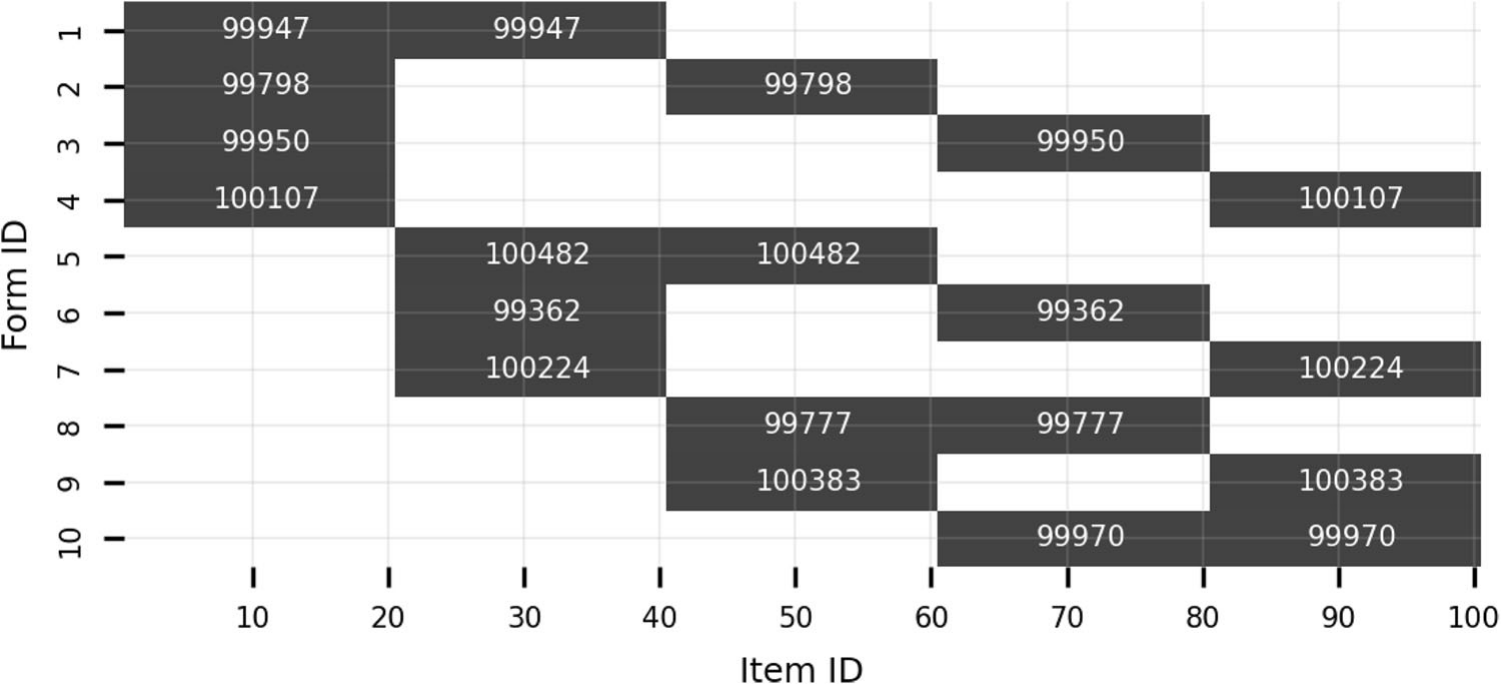
Study 3: Structure of item response data. *Note.* Each *shaded box* denotes a 20-item block. The texts note the number of non-missing responses

Five 20-item blocks were first created, forming a 100-item pool. For brevity, the same 20 items were repeated across blocks. Table 7 presents these items’ true parameter values. Next, ten 40-item forms were constructed by randomly selecting two of the five blocks. Each form contained 40 items, and every two forms had a 20-item overlap. One million respondents were then randomly assigned to one of the ten 40-item forms. Figure 6 shows the data structure, with the number of non-missing responses noted for each 20-item block.

**Table 7:** Study 3: True item parameters

Item	A	C		Item	A	C
1	0.66	$-0.18$		11	1.30	$-2.07$
2	0.76	0.14		12	1.30	1.26
3	0.89	$-0.05$		13	1.37	0.90
4	0.89	0.80		14	1.44	$-1.08$
5	0.97	1.22		15	1.47	0.09
6	1.04	$-0.74$		16	1.81	0.34
7	1.18	$-0.70$		17	1.83	$-2.20$
8	1.24	$-0.08$		18	2.07	0.64
9	1.26	0.09		19	2.36	$-0.21$
10	1.30	0.00		20	3.19	$-2.49$

*Note.* $B = -C/A$. B values (item difficulties) range between -1.26 and 1.59

**Table 8:** Study 3: Time to convergence in seconds

		0.1 Threshold			0.5 Threshold			0.9 Threshold	
Subset %		Time	Change %		Time	Change %		Time	Change %
10		387.89	- 60.78		442.02	- 55.30		530.96	- 46.31
20		477.93	- 51.67		532.52	- 46.15		620.40	- 37.27
30		514.33	- 47.99		569.07	- 42.46		658.46	- 33.42
50		696.10	- 29.61		750.36	- 24.13		838.97	- 15.17

*Note.* Standard EM (100% subset) takes 988.97 seconds

#### Evaluation metrics

##### Time to convergence

Aside from recording the runtime in seconds, we also computed % time change. The formula follows what has been discussed in Section “Evaluation metrics”.

##### Parameter recovery

30 $$\begin{aligned} \text {Mean Bias}\left( \hat{\phi }\right)&= \frac{1}{B}\sum _{b=1}^{B}\left( {\hat{\phi }}_b-\phi \right) \end{aligned}$$

where $\phi$ is the true parameter value and ${\hat{\phi }}_b$ is the estimate from Block *b* ($b = 1,\dots ,5$). In this case, because the same 20-item block is repeated five times to form the 100-item pool, we tracked the mean bias value for each parameter, along with the minimum and maximum values observed across its five repeated instances.

#### Results

##### Reduction in time to convergence

Table 8 summarizes convergence time (or runtime) in seconds.

##### Parameter recovery

**Fig. 7:**
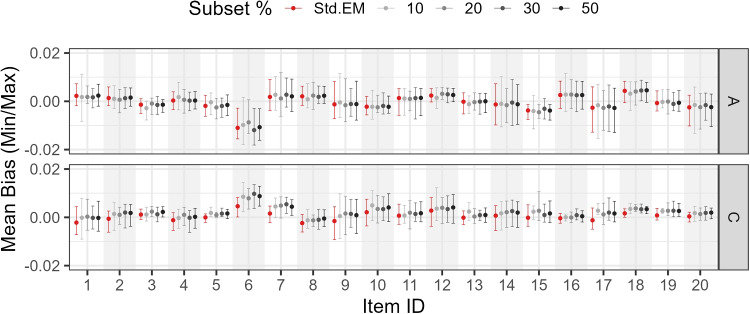
Study 3 (0.1 threshold): Mean bias by subset size, parameter type, and item

**Fig. 8:**
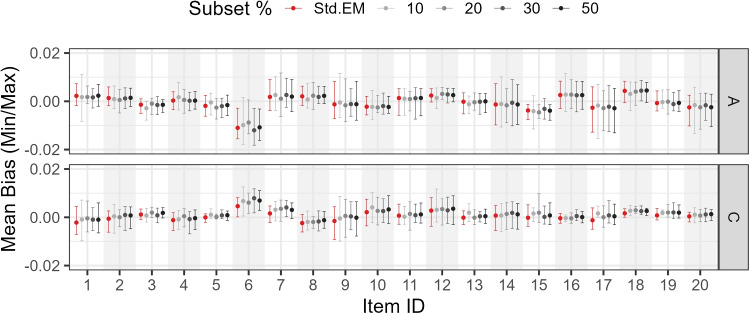
Study 3 (0.5 threshold): Mean bias by subset size, parameter type, and item

**Fig. 9:**
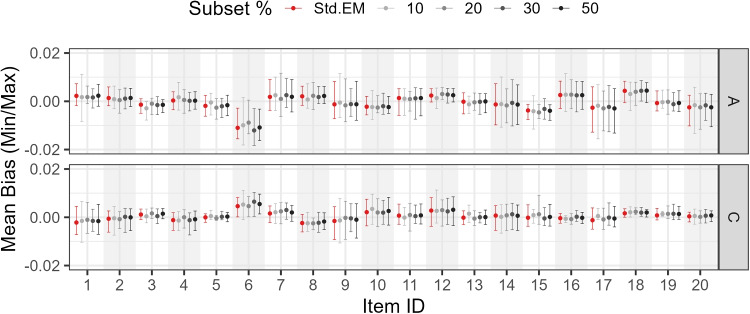
Study 3 (0.9 threshold): Mean bias by subset size, parameter type, and item

Figures 7, 8, and 9 show the mean, minimum, and maximum bias values for each parameter by subset size, parameter type, and item.

### Simulation 4: performance under departures from data unidimensionality

Study 4 served as a robustness check under less ideal data conditions. We stress-tested the modified algorithm in comparison with standard EM, and evaluated its performance in scenarios where a unidimensional IRT model may still be applied under a mild departure from unidimensionality (e.g., Ip, 2010). In such cases, data were generated from a multidimensional model, but estimation proceeded assuming unidimensionality. Greater departures from unidimensionality are expected to produce a less regular log-likelihood surface, deviating from the shape observed under a correctly specified unidimensional model, and to induce bias in item parameter estimates.

We used the testlet 2PL model as the data-generating model, where the latent variables were uncorrelated, and each item had equal slopes on the general dimension and a secondary, testlet-specific dimension. We fixed the variance of the general variable at 1.00 and constrained testlet variances to be equal, allowing the degree of data multidimensionality to be manipulated by varying only a common testlet variance.

#### Design

Study 4 varied two factors: (a) percentage subset size (10%, 20%, 30%, 50%, or 100% of the sample size) and (b) testlet variance (0.05, 0.10, 0.20, 0.50, 0.80, and 1.00). The number of testlets was fixed at three, with ten items per testlet, to introduce a stronger departure from unidimensionality when the testlet variance increased, compared with an alternative setup where the same total number of items was distributed over a larger number of smaller testlets.

Each condition was replicated 100 times; all replications were run until convergence. For each replication, binary responses were simulated for 60,000 individuals under a testlet 2PL model with the chosen testlet variance. Hyperparameters not varied in the design were held at their default values. Table 9 presents the true parameter values used.

#### Evaluation metric

The metric of interest was the per-parameter bias computed as in Eq. 26.

#### Results

This simulation served as a robustness check aimed at evaluating algorithm performance under different degrees of departure from data unidimensionality and less well-behaved log-likelihood surfaces. Across simulation conditions, the modified algorithm showed performance comparable to that of standard EM and did not introduce additional sensitivity to data multidimensionality.

**Fig. 10:**
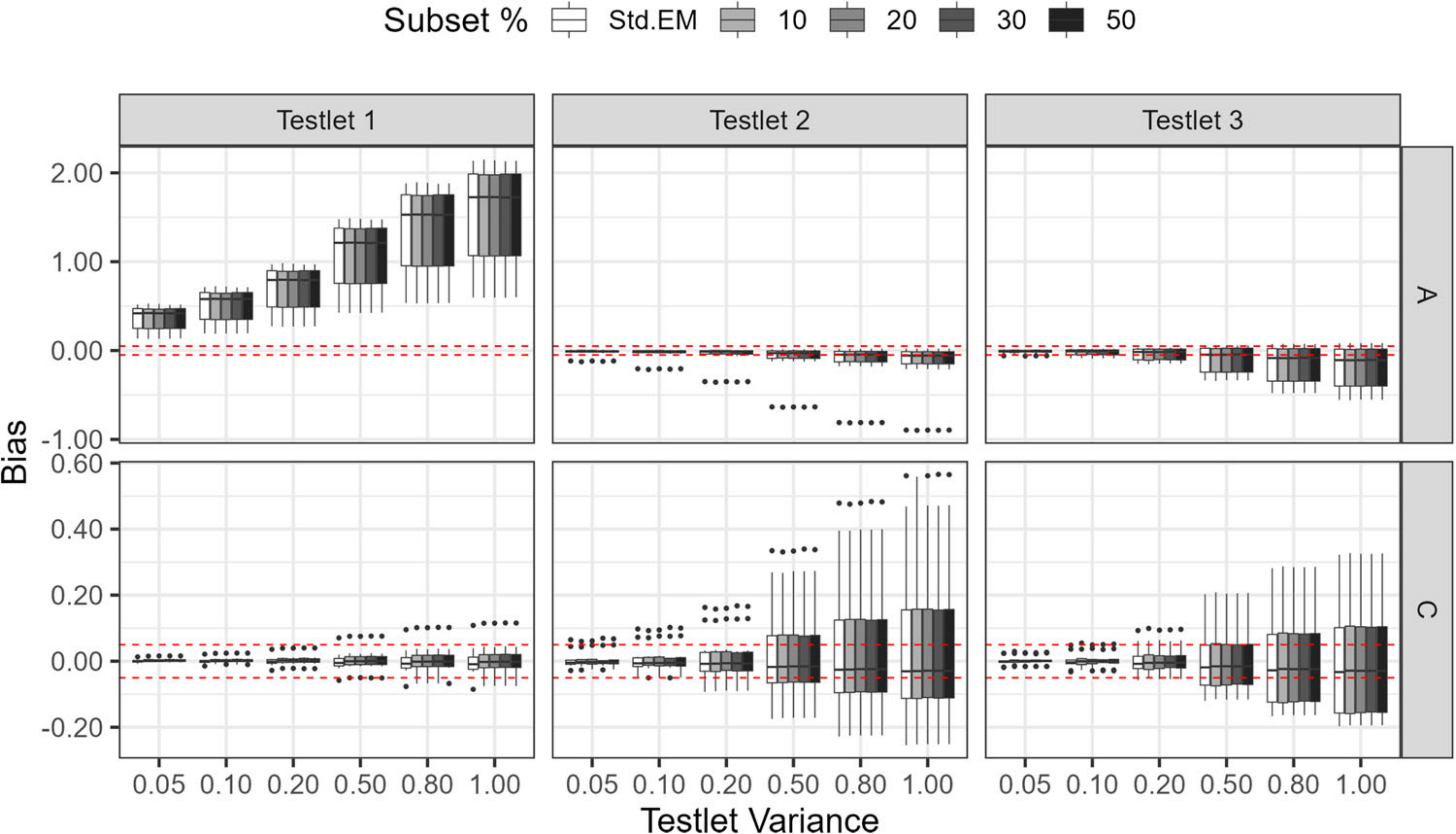
Study 4: Bias for parameter estimates. *Note.* *Red lines* mark the $\pm 0.05$ levels

Figure 10 shows patterns of parameter bias, with the $\pm 0.05$ levels highlighted in red for reference. As testlet variance increased, a greater departure from unidimensionality occurred, and hence bias increased. Slope (A) parameters were much more affected by multidimensionality than intercept (C) parameters. This result was consistent with past observations noting that estimation bias was more substantial for item slopes than for item difficulty or intercept parameters (Wainer & Wang, 2001). High absolute bias was associated with items whose true slopes exceeded 2.0 (e.g., Item 1 and Item 15) or that belonged to a high-slope cluster (e.g., Testlet 1). Within a testlet, strongly discriminating items, or items with higher slopes, anchored the testlet dimension, making the unmodeled testlet effect more influential and increasing distortion for neighboring items despite their lower slopes. This was clear when we compared the results between Testlet 1 and the other testlets.

**Table 9:** Study 4: True item parameters

Testlet 1				Testlet 2				Testlet 3		
Item	A	C		Item	A	C		Item	A	C
1	2.49	$-0.16$		11	1.28	0.57		21	0.91	2.17
2	1.26	0.14		12	0.96	0.48		22	1.66	0.21
3	2.15	0.21		13	0.83	1.08		23	1.68	0.84
4	2.17	0.16		14	0.81	$-2.23$		24	2.28	0.57
5	2.45	0.78		15	2.45	0.61		25	0.72	1.80
6	0.65	$-0.13$		16	1.35	$-2.41$		26	1.49	$-0.03$
7	1.13	0.55		17	0.63	$-0.06$		27	1.08	$-1.14$
8	2.34	0.30		18	1.48	$-2.48$		28	1.47	$-1.19$
9	1.85	$-1.12$		19	1.08	1.35		29	2.40	$-0.92$
10	1.07	$-0.92$		20	0.92	$-0.86$		30	2.35	0.05

*Note.* Each item has the same slope value (A) for the general and testlet latent variables

## Discussion

### Summary of findings

In this paper, we proposed a modified EM algorithm to accelerate convergence in large-scale data settings, with simulation studies conducted on item counts ranging from 18 to 100 (item pool) and sample sizes of 60,000 and 1 million. From the simulations, we observed the following patterns. First, runtime reductions achieved by the modified algorithm scaled with item count, with the largest reduction (60%) observed in Study 3, which simulated a large-scale testing scenario with 1 million respondents completing 40-item forms assembled from a 100-item pool. Second, slope parameters showed better recovery—lower bias and RMSE—than intercepts, and parameters with medium true values showed better recovery than those with extreme values (see Section “Grid sampling of true item parameters” for definitions). Third, standard error estimates had absolute bias below 0.01 and RMSE below 0.02 across conditions. Fourth, estimation bias and RMSE under the modified algorithm approached those of standard EM with larger subset sizes and higher transition thresholds. However, as sample size increases, smaller subset sizes and thresholds can achieve comparable performance, as demonstrated in Study 3 (Section “Simulation 3: A large-scale form-based testing scenario”). Overall, the modified algorithm reduced time to convergence while maintaining comparable estimation performance under larger item counts.

Specifically, the modified algorithm achieved consistent time savings across all subset sizes when the item count was moderate to large, such as 54, but not when the item count was 18. This difference likely reflects how the structure of the response pattern matrix and the runtime of IRT EM estimation scale with the number of items, given a fixed sample size. With more items, individuals are more likely to produce unique response patterns, thereby increasing the size of the pattern matrix. In such cases, enumerating over response patterns becomes as costly as processing the full item data, making the subset-based approach more efficient. In contrast, the pattern matrix remains compact with 18 items, and the overhead of managing subsets and subsampling variability can outweigh the computational benefits.

### Implications for future work

Our work highlights several directions for future research and application. Standard EM-based IRT estimation remains widely used in large-scale assessments and certification exams. One example is the SAT, which collects item-level data from millions of examinees annually (College Board, 2025). In such contexts, the computational burden of full-data EM estimation for item calibration can be substantial. The modified algorithm provides an alternative that reduces runtime while achieving comparable estimation performance.

Another strength of this work is that the proposed modifications can be integrated into existing IRT pipelines with minimal changes to core procedures. Future work could explore packaging them as modular add-ons to existing software to support adoption in operational contexts. Empirical validation using real-world data could also help identify practical limits and evaluate trade-offs between efficiency, accuracy, and precision.

We obtained these results under a unidimensional model and specific simulation conditions. Future research could examine whether the modified algorithm and its variants extend to multidimensional IRT settings. This includes investigating whether selectively subsetting by item clusters or examinee groups yields similar or greater computational benefits, given that the number of quadrature nodes evaluated during the E-step increases exponentially with the number of latent variables. Another research direction is to explore parallel computing and ensemble-styled extensions by running multiple independent instances of the modified EM algorithm, or a combination of the standard and modified algorithms, in parallel with relaxed convergence criteria. This approach differs from parallelizing a single run under a conventional fork-and-join paradigm. Results are then aggregated across instances to mitigate the impact of idiosyncratic error from any single run, assess stability, and reduce overall runtime.

## Supplementary Information

Below is the link to the electronic supplementary material.

Supplementary file 1 (pdf 66 KB)

## Figures and Tables

**Algorithm 1:**
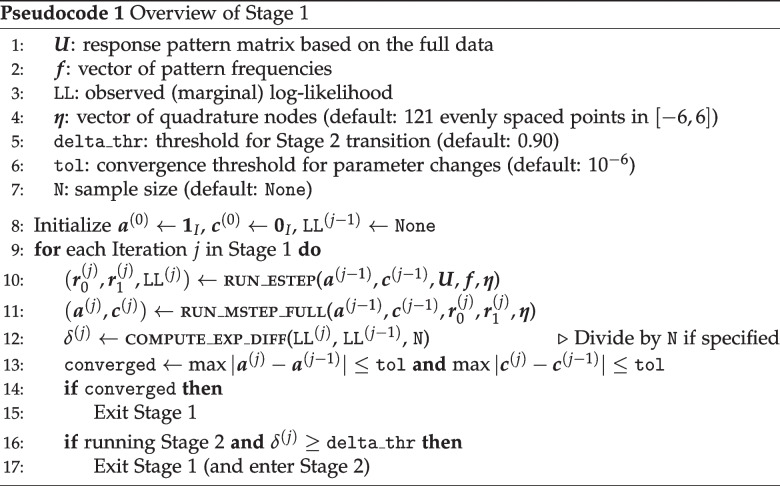
Overview of Stage 1

**Algorithm 2:**
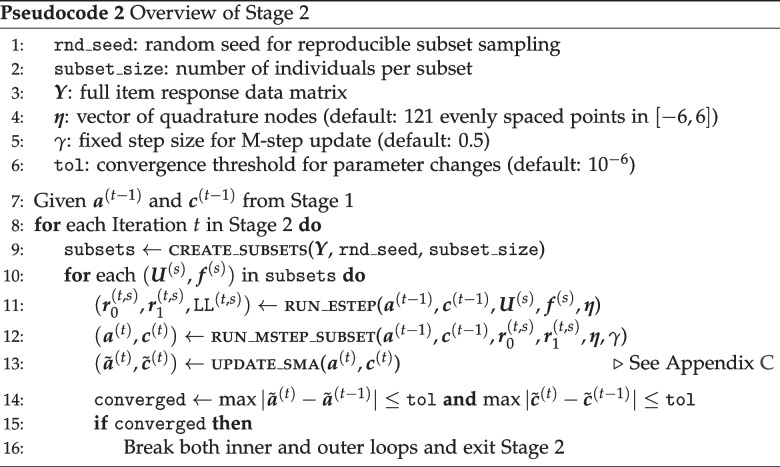
Overview of Stage 2

**Algorithm 3:**
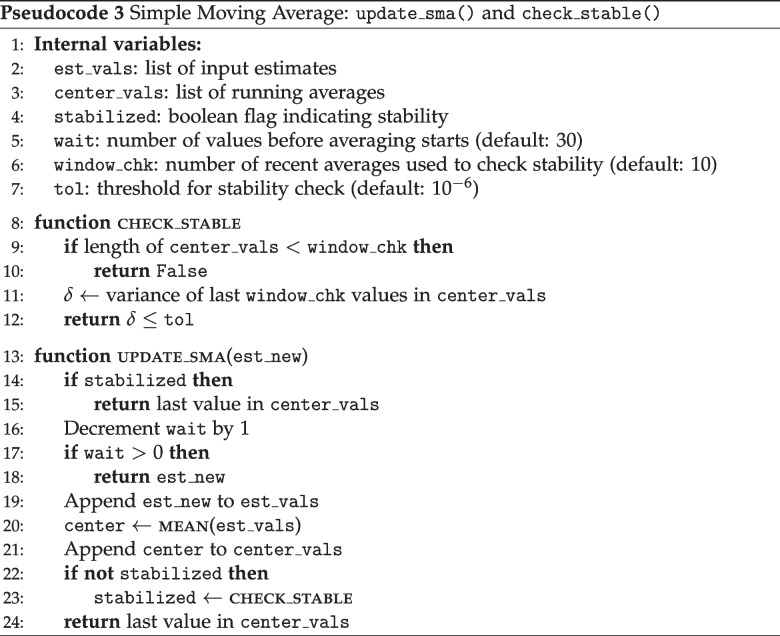
Simple Moving Average: update_sma() and check_stable()

**Algorithm 4:**
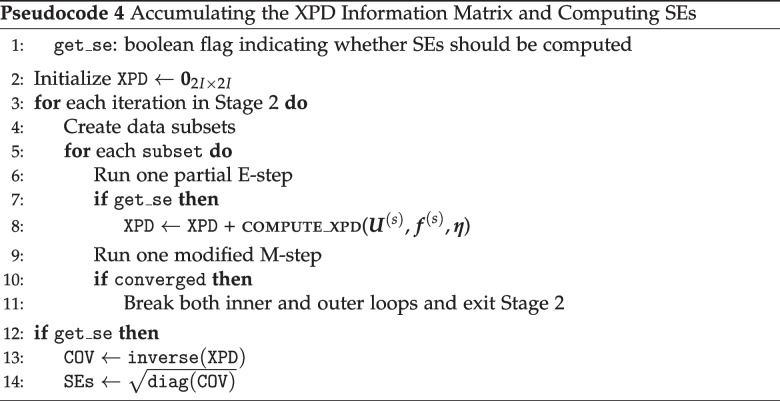
Accumulating the XPD Information Matrix and Computing SEs

## Data Availability

This research did not involve the collection or analysis of any empirical data or materials. Detailed results from simulation studies are included in Supplemental Files at https://github.com/teannafeng/paper-brm-modified-em.

## Footnotes

1 Equation 13 is equivalent to taking a step that minimizes the negative log-likelihood, which is the default behavior for optimizers such as nlm() or optim() in R and scipy.optimize.minimize() in Python.

2 With numba (v0.61.2), numpy (v2.2.3), and scipy (v1.15.2).

3 Values in Table 5 and later tables are shown with rounding; minor differences may occur. Tables for the 36-item condition are omitted here for brevity.

## Code Availability

Code is available at https://github.com/teannafeng/paper-brm-modified-em.
