# Supplementary material for: A modified expectation–maximization algorithm for accelerated item response theory model estimation with large datasets
Source: Behav Res Methods. 2026 Apr 21;58(5):133. doi: 10.3758/s13428-026-02996-0 (PMC13099681; doi:10.3758/s13428-026-02996-0)
Supplement: Supplementary file 1 — (pdf 66 KB) [file 13428_2026_2996_MOESM1_ESM.pdf]

Supplemental Files
Simulation 1: By-Parameter Results

Table S1. Study 1 (10% Subset Size + 54 Items + 0.90 Threshold): Bias and RMSE by Parameter

	Parameter A					Parameter C				
Item	True value	M_{est}	SD_{est}	Bias	RMSE	True value	M_{est}	SD_{est}	Bias	RMSE
1	0.64	0.63	0.01	0.00	0.01	1.06	1.06	0.01	0.00	0.01
2	1.43	1.42	0.02	−0.01	0.03	3.34	3.33	0.03	−0.01	0.03
3	0.89	0.89	0.02	0.00	0.02	2.32	2.32	0.02	0.00	0.02
4	1.16	1.16	0.02	0.00	0.02	2.62	2.61	0.02	−0.01	0.02
5	1.46	1.46	0.02	0.00	0.02	2.63	2.63	0.02	−0.01	0.02
6	0.96	0.95	0.01	0.00	0.01	1.72	1.71	0.01	−0.01	0.01
7	0.54	0.54	0.01	0.00	0.01	−0.44	−0.44	0.01	0.00	0.01
8	0.50	0.51	0.01	0.00	0.01	0.31	0.31	0.01	0.00	0.01
9	1.11	1.11	0.02	0.00	0.02	−1.38	−1.38	0.02	0.00	0.02
10	0.80	0.80	0.01	0.00	0.01	0.60	0.60	0.01	0.00	0.01
11	1.17	1.17	0.02	0.00	0.02	−1.71	−1.71	0.02	0.00	0.02
12	0.97	0.97	0.01	0.00	0.01	1.09	1.09	0.01	0.00	0.01
13	1.42	1.41	0.03	0.00	0.03	−4.13	−4.13	0.04	0.01	0.04
14	0.78	0.77	0.01	0.00	0.02	−1.77	−1.77	0.02	0.00	0.02
15	0.65	0.66	0.01	0.00	0.01	−1.00	−1.00	0.01	0.00	0.01
16	0.82	0.83	0.02	0.00	0.02	−2.46	−2.46	0.02	0.00	0.02
17	1.01	1.02	0.02	0.00	0.02	−2.85	−2.86	0.02	0.00	0.02
18	0.57	0.57	0.01	0.00	0.01	−1.09	−1.09	0.01	0.00	0.01
19	1.97	1.97	0.03	0.00	0.03	3.66	3.65	0.03	0.00	0.03
20	2.42	2.42	0.05	0.00	0.05	5.84	5.83	0.08	−0.01	0.08
21	2.43	2.41	0.04	−0.02	0.05	5.47	5.44	0.07	−0.03	0.08
22	2.30	2.30	0.03	0.00	0.03	3.81	3.80	0.04	−0.01	0.04
23	1.98	1.98	0.03	0.00	0.03	3.43	3.42	0.03	0.00	0.03
24	1.78	1.78	0.03	0.00	0.03	4.42	4.42	0.05	0.00	0.05
25	1.61	1.60	0.02	0.00	0.02	−0.11	−0.12	0.01	0.00	0.01
26	2.15	2.15	0.03	0.00	0.03	−2.58	−2.58	0.02	0.00	0.02
27	1.60	1.61	0.02	0.01	0.02	0.91	0.90	0.01	0.00	0.01
28	1.58	1.58	0.02	0.00	0.02	−2.07	−2.08	0.02	0.00	0.02
29	2.46	2.46	0.03	0.00	0.03	2.28	2.27	0.03	−0.01	0.03
30	2.45	2.46	0.02	0.01	0.03	0.41	0.41	0.02	0.00	0.02
31	1.76	1.76	0.03	0.00	0.03	−4.42	−4.42	0.04	−0.01	0.05
32	1.88	1.88	0.05	0.00	0.05	−5.59	−5.59	0.08	0.00	0.08
33	2.17	2.18	0.06	0.01	0.06	−6.47	−6.49	0.10	−0.02	0.10
34	2.47	2.48	0.04	0.00	0.04	−4.38	−4.39	0.05	0.00	0.05
35	1.94	1.93	0.04	−0.01	0.04	−4.87	−4.87	0.05	0.01	0.05
36	2.15	2.16	0.04	0.00	0.04	−4.47	−4.47	0.05	0.00	0.05
37	3.17	3.17	0.05	0.00	0.05	6.09	6.07	0.07	−0.01	0.07
38	3.28	3.27	0.09	−0.02	0.09	8.27	8.23	0.17	−0.04	0.17
39	2.60	2.60	0.05	0.00	0.05	4.54	4.54	0.06	0.00	0.06
40	2.53	2.52	0.06	0.00	0.06	6.67	6.66	0.10	−0.01	0.10
41	3.36	3.33	0.12	−0.02	0.12	9.82	9.75	0.25	−0.07	0.26
42	2.69	2.70	0.06	0.00	0.06	6.16	6.15	0.09	−0.01	0.09
43	2.74	2.74	0.03	0.00	0.03	1.16	1.15	0.02	0.00	0.02
44	2.65	2.64	0.03	0.00	0.03	2.89	2.88	0.03	−0.01	0.03
45	2.60	2.60	0.03	0.00	0.03	2.71	2.71	0.03	−0.01	0.03
46	2.64	2.64	0.03	0.00	0.03	3.73	3.73	0.04	0.00	0.04
47	3.23	3.23	0.04	0.00	0.04	−1.71	−1.71	0.03	0.00	0.03
48	2.94	2.95	0.04	0.00	0.04	3.31	3.31	0.04	−0.01	0.04
49	2.68	2.67	0.06	−0.01	0.06	−6.46	−6.44	0.10	0.02	0.10
50	2.77	2.76	0.07	−0.01	0.07	−7.12	−7.10	0.13	0.02	0.13
51	2.87	2.87	0.05	0.00	0.05	−5.50	−5.50	0.07	0.00	0.07
52	3.38	3.39	0.07	0.01	0.07	−5.92	−5.93	0.10	−0.01	0.10
53	2.60	2.60	0.08	0.00	0.08	−7.70	−7.69	0.17	0.01	0.17
54	3.46	3.45	0.06	−0.01	0.06	−5.83	−5.82	0.09	0.01	0.09

Table S2. Study 1 (20% Subset Size + 54 Items + 0.90 Threshold): Bias and RMSE by Parameter

	Parameter A					Parameter C				
Item	True value	M_{est}	SD_{est}	Bias	RMSE	True value	M_{est}	SD_{est}	Bias	RMSE
1	0.64	0.63	0.01	0.00	0.01	1.06	1.06	0.01	0.00	0.01
2	1.43	1.43	0.02	0.00	0.02	3.34	3.34	0.03	0.00	0.03
3	0.89	0.89	0.02	0.00	0.02	2.32	2.32	0.02	0.00	0.02
4	1.16	1.16	0.02	0.00	0.02	2.62	2.62	0.02	0.00	0.02
5	1.46	1.46	0.02	0.00	0.02	2.63	2.63	0.02	0.00	0.02
6	0.96	0.96	0.01	0.00	0.01	1.72	1.72	0.01	0.00	0.01
7	0.54	0.54	0.01	0.00	0.01	−0.44	−0.44	0.01	0.00	0.01
8	0.50	0.50	0.01	0.00	0.01	0.31	0.31	0.01	0.00	0.01
9	1.11	1.11	0.01	0.00	0.01	−1.38	−1.38	0.01	0.00	0.01
10	0.80	0.80	0.01	0.00	0.01	0.60	0.60	0.01	0.00	0.01
11	1.17	1.17	0.02	0.00	0.02	−1.71	−1.71	0.02	0.00	0.02
12	0.97	0.97	0.01	0.00	0.01	1.09	1.09	0.01	0.00	0.01
13	1.42	1.41	0.03	0.00	0.03	−4.13	−4.13	0.04	0.01	0.04
14	0.78	0.78	0.01	0.00	0.01	−1.77	−1.77	0.02	0.00	0.02
15	0.65	0.65	0.01	0.00	0.01	−1.00	−1.00	0.01	0.00	0.01
16	0.82	0.82	0.02	0.00	0.02	−2.46	−2.46	0.02	0.00	0.02
17	1.01	1.02	0.02	0.00	0.02	−2.85	−2.86	0.02	0.00	0.02
18	0.57	0.57	0.01	0.00	0.01	−1.09	−1.09	0.01	0.00	0.01
19	1.97	1.97	0.03	0.00	0.03	3.66	3.66	0.03	0.00	0.03
20	2.42	2.42	0.05	0.00	0.05	5.84	5.83	0.07	−0.01	0.07
21	2.43	2.41	0.04	−0.02	0.05	5.47	5.44	0.07	−0.02	0.07
22	2.30	2.30	0.04	0.00	0.04	3.81	3.81	0.04	0.00	0.04
23	1.98	1.99	0.03	0.00	0.03	3.43	3.42	0.03	0.00	0.03
24	1.78	1.78	0.03	0.00	0.03	4.42	4.42	0.05	0.00	0.05
25	1.61	1.60	0.02	0.00	0.02	−0.11	−0.11	0.01	0.00	0.01
26	2.15	2.15	0.02	0.00	0.02	−2.58	−2.58	0.02	0.00	0.02
27	1.60	1.61	0.02	0.00	0.02	0.91	0.91	0.01	0.00	0.01
28	1.58	1.58	0.02	0.00	0.02	−2.07	−2.08	0.02	0.00	0.02
29	2.46	2.46	0.03	0.00	0.03	2.28	2.27	0.03	−0.01	0.03
30	2.45	2.46	0.02	0.01	0.02	0.41	0.41	0.02	0.00	0.02
31	1.76	1.76	0.03	0.00	0.03	−4.42	−4.42	0.04	−0.01	0.04
32	1.88	1.88	0.05	0.00	0.05	−5.59	−5.59	0.08	−0.01	0.08
33	2.17	2.18	0.06	0.01	0.06	−6.47	−6.49	0.10	−0.02	0.10
34	2.47	2.48	0.04	0.00	0.04	−4.38	−4.39	0.05	0.00	0.05
35	1.94	1.94	0.03	−0.01	0.03	−4.87	−4.87	0.05	0.00	0.05
36	2.15	2.15	0.04	0.00	0.04	−4.47	−4.47	0.05	0.00	0.05
37	3.17	3.17	0.05	0.00	0.05	6.09	6.07	0.07	−0.01	0.07
38	3.28	3.27	0.09	−0.01	0.09	8.27	8.24	0.17	−0.03	0.17
39	2.60	2.60	0.04	0.00	0.04	4.54	4.54	0.06	0.00	0.06
40	2.53	2.52	0.06	0.00	0.06	6.67	6.66	0.10	−0.01	0.10
41	3.36	3.34	0.11	−0.02	0.11	9.82	9.75	0.24	−0.06	0.25
42	2.69	2.70	0.06	0.00	0.06	6.16	6.15	0.09	−0.01	0.09
43	2.74	2.74	0.03	0.00	0.03	1.16	1.15	0.02	0.00	0.02
44	2.65	2.64	0.03	0.00	0.03	2.89	2.89	0.03	0.00	0.03
45	2.60	2.59	0.03	0.00	0.03	2.71	2.71	0.03	−0.01	0.03
46	2.64	2.64	0.03	0.00	0.03	3.73	3.73	0.03	0.00	0.03
47	3.23	3.23	0.04	0.00	0.04	−1.71	−1.71	0.02	0.00	0.02
48	2.94	2.95	0.04	0.00	0.04	3.31	3.31	0.04	0.00	0.04
49	2.68	2.66	0.06	−0.01	0.06	−6.46	−6.44	0.10	0.02	0.10
50	2.77	2.76	0.07	−0.01	0.07	−7.12	−7.10	0.12	0.02	0.12
51	2.87	2.87	0.05	0.00	0.05	−5.50	−5.50	0.07	0.00	0.07
52	3.38	3.39	0.06	0.01	0.06	−5.92	−5.93	0.09	−0.01	0.09
53	2.60	2.60	0.08	0.00	0.08	−7.70	−7.70	0.17	0.00	0.17
54	3.46	3.45	0.06	−0.01	0.06	−5.83	−5.82	0.09	0.01	0.09

Table S3. Study 1 (30% Subset Size + 54 Items + 0.90 Threshold): Bias and RMSE by Parameter

	Parameter A					Parameter C				
Item	True value	M_{est}	SD_{est}	Bias	RMSE	True value	M_{est}	SD_{est}	Bias	RMSE
1	0.64	0.63	0.01	0.00	0.01	1.06	1.05	0.01	−0.01	0.01
2	1.43	1.43	0.02	0.00	0.02	3.34	3.34	0.03	0.00	0.03
3	0.89	0.89	0.02	0.00	0.02	2.32	2.32	0.02	0.00	0.02
4	1.16	1.16	0.02	0.00	0.02	2.62	2.62	0.02	0.00	0.02
5	1.46	1.47	0.02	0.00	0.02	2.63	2.63	0.02	0.00	0.02
6	0.96	0.95	0.01	0.00	0.01	1.72	1.72	0.01	0.00	0.01
7	0.54	0.54	0.01	0.00	0.01	−0.44	−0.44	0.01	0.00	0.01
8	0.50	0.50	0.01	0.00	0.01	0.31	0.31	0.01	0.00	0.01
9	1.11	1.11	0.01	0.00	0.01	−1.38	−1.38	0.01	0.00	0.01
10	0.80	0.80	0.01	0.00	0.01	0.60	0.60	0.01	0.00	0.01
11	1.17	1.17	0.01	0.00	0.01	−1.71	−1.71	0.01	0.00	0.01
12	0.97	0.97	0.01	0.00	0.01	1.09	1.09	0.01	0.00	0.01
13	1.42	1.41	0.03	0.00	0.03	−4.13	−4.13	0.04	0.01	0.04
14	0.78	0.78	0.01	0.00	0.01	−1.77	−1.77	0.01	0.00	0.01
15	0.65	0.65	0.01	0.00	0.01	−1.00	−1.00	0.01	0.00	0.01
16	0.82	0.83	0.01	0.00	0.01	−2.46	−2.46	0.02	0.00	0.02
17	1.01	1.02	0.02	0.00	0.02	−2.85	−2.85	0.02	0.00	0.02
18	0.57	0.57	0.01	0.00	0.01	−1.09	−1.09	0.01	0.00	0.01
19	1.97	1.97	0.03	0.00	0.03	3.66	3.66	0.03	0.00	0.03
20	2.42	2.42	0.05	0.00	0.05	5.84	5.83	0.08	−0.01	0.08
21	2.43	2.41	0.04	−0.01	0.05	5.47	5.45	0.07	−0.02	0.07
22	2.30	2.30	0.03	0.00	0.03	3.81	3.81	0.04	0.00	0.04
23	1.98	1.99	0.03	0.00	0.03	3.43	3.42	0.03	0.00	0.03
24	1.78	1.78	0.03	0.00	0.03	4.42	4.42	0.04	0.00	0.04
25	1.61	1.60	0.02	0.00	0.02	−0.11	−0.12	0.01	0.00	0.01
26	2.15	2.15	0.02	0.01	0.03	−2.58	−2.58	0.02	0.00	0.02
27	1.60	1.61	0.02	0.00	0.02	0.91	0.91	0.01	0.00	0.01
28	1.58	1.58	0.02	0.00	0.02	−2.07	−2.07	0.02	0.00	0.02
29	2.46	2.46	0.03	0.00	0.03	2.28	2.27	0.03	−0.01	0.03
30	2.45	2.46	0.02	0.01	0.03	0.41	0.41	0.02	0.00	0.02
31	1.76	1.76	0.03	0.00	0.03	−4.42	−4.42	0.04	−0.01	0.04
32	1.88	1.88	0.05	0.00	0.05	−5.59	−5.59	0.08	−0.01	0.08
33	2.17	2.18	0.06	0.01	0.06	−6.47	−6.49	0.10	−0.02	0.10
34	2.47	2.48	0.04	0.00	0.04	−4.38	−4.38	0.05	0.00	0.05
35	1.94	1.94	0.03	−0.01	0.03	−4.87	−4.87	0.05	0.01	0.05
36	2.15	2.15	0.04	0.00	0.04	−4.47	−4.47	0.05	0.00	0.05
37	3.17	3.17	0.05	0.00	0.05	6.09	6.08	0.07	−0.01	0.07
38	3.28	3.27	0.09	−0.01	0.09	8.27	8.24	0.17	−0.03	0.17
39	2.60	2.60	0.04	0.00	0.04	4.54	4.54	0.06	0.00	0.06
40	2.53	2.52	0.06	0.00	0.06	6.67	6.66	0.10	−0.01	0.10
41	3.36	3.34	0.12	−0.02	0.12	9.82	9.76	0.25	−0.05	0.26
42	2.69	2.70	0.06	0.00	0.06	6.16	6.15	0.09	−0.01	0.09
43	2.74	2.74	0.03	0.00	0.03	1.16	1.15	0.02	0.00	0.02
44	2.65	2.65	0.03	0.00	0.03	2.89	2.89	0.03	0.00	0.03
45	2.60	2.60	0.03	0.00	0.03	2.71	2.71	0.03	0.00	0.03
46	2.64	2.64	0.03	0.00	0.03	3.73	3.73	0.03	0.00	0.03
47	3.23	3.23	0.04	0.00	0.04	−1.71	−1.71	0.02	0.00	0.02
48	2.94	2.95	0.04	0.00	0.04	3.31	3.31	0.04	0.00	0.04
49	2.68	2.67	0.06	−0.01	0.06	−6.46	−6.44	0.10	0.02	0.10
50	2.77	2.76	0.07	−0.01	0.07	−7.12	−7.10	0.12	0.02	0.12
51	2.87	2.87	0.04	0.00	0.04	−5.50	−5.50	0.07	0.00	0.06
52	3.38	3.39	0.06	0.00	0.06	−5.92	−5.92	0.09	−0.01	0.09
53	2.60	2.60	0.08	0.00	0.08	−7.70	−7.70	0.17	0.01	0.17
54	3.46	3.45	0.06	−0.01	0.06	−5.83	−5.82	0.09	0.02	0.09

Table S4. Study 1 (50% Subset Size + 54 Items + 0.90 Threshold): Bias and RMSE by Parameter

	Parameter A					Parameter C				
Item	True value	M_{est}	SD_{est}	Bias	RMSE	True value	M_{est}	SD_{est}	Bias	RMSE
1	0.64	0.63	0.01	0.00	0.01	1.06	1.06	0.01	0.00	0.01
2	1.43	1.43	0.02	0.00	0.02	3.34	3.33	0.03	−0.01	0.03
3	0.89	0.89	0.02	0.00	0.02	2.32	2.32	0.02	0.00	0.02
4	1.16	1.16	0.02	0.00	0.02	2.62	2.61	0.02	0.00	0.02
5	1.46	1.46	0.02	0.00	0.02	2.63	2.63	0.02	0.00	0.02
6	0.96	0.96	0.01	0.00	0.01	1.72	1.72	0.01	0.00	0.01
7	0.54	0.54	0.01	0.00	0.01	−0.44	−0.44	0.01	0.00	0.01
8	0.50	0.50	0.01	0.00	0.01	0.31	0.31	0.01	0.00	0.01
9	1.11	1.11	0.01	0.00	0.01	−1.38	−1.38	0.01	0.00	0.01
10	0.80	0.80	0.01	0.00	0.01	0.60	0.60	0.01	0.00	0.01
11	1.17	1.17	0.02	0.00	0.02	−1.71	−1.71	0.01	0.00	0.01
12	0.97	0.97	0.01	0.00	0.01	1.09	1.09	0.01	0.00	0.01
13	1.42	1.41	0.03	0.00	0.03	−4.13	−4.13	0.04	0.01	0.04
14	0.78	0.78	0.01	0.00	0.01	−1.77	−1.77	0.01	0.00	0.01
15	0.65	0.65	0.01	0.00	0.01	−1.00	−1.00	0.01	0.00	0.01
16	0.82	0.82	0.01	0.00	0.01	−2.46	−2.46	0.02	0.00	0.02
17	1.01	1.02	0.02	0.00	0.02	−2.85	−2.85	0.02	0.00	0.02
18	0.57	0.57	0.01	0.00	0.01	−1.09	−1.09	0.01	0.00	0.01
19	1.97	1.97	0.03	0.00	0.03	3.66	3.66	0.03	0.00	0.03
20	2.42	2.42	0.05	0.00	0.05	5.84	5.83	0.07	−0.01	0.07
21	2.43	2.41	0.04	−0.01	0.05	5.47	5.45	0.07	−0.02	0.07
22	2.30	2.30	0.03	0.00	0.03	3.81	3.81	0.04	0.00	0.04
23	1.98	1.99	0.03	0.00	0.03	3.43	3.42	0.03	0.00	0.03
24	1.78	1.78	0.03	0.00	0.03	4.42	4.42	0.05	0.00	0.05
25	1.61	1.60	0.02	0.00	0.02	−0.11	−0.11	0.01	0.00	0.01
26	2.15	2.15	0.02	0.00	0.02	−2.58	−2.58	0.02	0.00	0.02
27	1.60	1.61	0.02	0.00	0.02	0.91	0.91	0.01	0.00	0.01
28	1.58	1.58	0.02	0.00	0.02	−2.07	−2.08	0.02	0.00	0.02
29	2.46	2.46	0.03	0.00	0.03	2.28	2.27	0.03	−0.01	0.03
30	2.45	2.46	0.02	0.01	0.02	0.41	0.41	0.02	0.00	0.02
31	1.76	1.76	0.03	0.00	0.03	−4.42	−4.42	0.04	−0.01	0.04
32	1.88	1.88	0.05	0.00	0.05	−5.59	−5.60	0.08	−0.01	0.08
33	2.17	2.18	0.06	0.01	0.06	−6.47	−6.49	0.10	−0.02	0.10
34	2.47	2.48	0.04	0.00	0.04	−4.38	−4.39	0.05	0.00	0.05
35	1.94	1.93	0.03	−0.01	0.03	−4.87	−4.87	0.05	0.00	0.05
36	2.15	2.15	0.04	0.00	0.04	−4.47	−4.47	0.05	0.00	0.05
37	3.17	3.17	0.05	0.00	0.05	6.09	6.08	0.07	−0.01	0.07
38	3.28	3.27	0.09	−0.01	0.09	8.27	8.24	0.17	−0.03	0.17
39	2.60	2.60	0.04	0.00	0.04	4.54	4.54	0.06	0.00	0.06
40	2.53	2.52	0.06	0.00	0.06	6.67	6.67	0.10	−0.01	0.10
41	3.36	3.34	0.11	−0.01	0.11	9.82	9.77	0.25	−0.05	0.25
42	2.69	2.70	0.06	0.00	0.06	6.16	6.16	0.09	0.00	0.09
43	2.74	2.74	0.03	0.00	0.03	1.16	1.15	0.02	0.00	0.02
44	2.65	2.64	0.03	0.00	0.03	2.89	2.89	0.03	0.00	0.03
45	2.60	2.60	0.03	0.00	0.03	2.71	2.71	0.03	0.00	0.03
46	2.64	2.64	0.03	0.00	0.03	3.73	3.73	0.03	0.00	0.03
47	3.23	3.23	0.04	0.00	0.04	−1.71	−1.71	0.02	0.00	0.02
48	2.94	2.95	0.03	0.00	0.03	3.31	3.31	0.04	0.00	0.04
49	2.68	2.67	0.06	−0.01	0.06	−6.46	−6.44	0.10	0.02	0.10
50	2.77	2.76	0.07	−0.01	0.07	−7.12	−7.10	0.12	0.02	0.12
51	2.87	2.87	0.05	0.00	0.04	−5.50	−5.51	0.06	0.00	0.06
52	3.38	3.39	0.06	0.01	0.06	−5.92	−5.93	0.09	−0.01	0.09
53	2.60	2.60	0.08	0.00	0.08	−7.70	−7.70	0.17	0.00	0.17
54	3.46	3.45	0.06	−0.01	0.06	−5.83	−5.82	0.09	0.01	0.09

Table S5. Study 1 (100% Subset Size + 54 Items): Bias and RMSE by Parameter

	Parameter A					Parameter C				
Item	True value	M_{est}	SD_{est}	Bias	RMSE	True value	M_{est}	SD_{est}	Bias	RMSE
1	0.64	0.64	0.01	0.00	0.01	1.06	1.06	0.01	0.00	0.01
2	1.43	1.43	0.02	0.00	0.02	3.34	3.34	0.03	0.00	0.03
3	0.89	0.89	0.02	0.00	0.02	2.32	2.32	0.02	0.00	0.02
4	1.16	1.16	0.02	0.00	0.02	2.62	2.62	0.02	0.00	0.02
5	1.46	1.46	0.02	0.00	0.02	2.63	2.63	0.02	0.00	0.02
6	0.96	0.96	0.01	0.00	0.01	1.72	1.72	0.01	0.00	0.01
7	0.54	0.54	0.01	0.00	0.01	−0.44	−0.44	0.01	0.00	0.01
8	0.50	0.50	0.01	0.00	0.01	0.31	0.31	0.01	0.00	0.01
9	1.11	1.11	0.01	0.00	0.01	−1.38	−1.38	0.01	0.00	0.01
10	0.80	0.80	0.01	0.00	0.01	0.60	0.60	0.01	0.00	0.01
11	1.17	1.17	0.01	0.00	0.01	−1.71	−1.71	0.01	0.00	0.01
12	0.97	0.97	0.01	0.00	0.01	1.09	1.09	0.01	0.00	0.01
13	1.42	1.41	0.03	0.00	0.03	−4.13	−4.13	0.04	0.01	0.04
14	0.78	0.78	0.01	0.00	0.01	−1.77	−1.77	0.01	0.00	0.01
15	0.65	0.65	0.01	0.00	0.01	−1.00	−1.00	0.01	0.00	0.01
16	0.82	0.82	0.01	0.00	0.01	−2.46	−2.46	0.02	0.00	0.02
17	1.01	1.02	0.02	0.00	0.02	−2.85	−2.85	0.02	0.00	0.02
18	0.57	0.57	0.01	0.00	0.01	−1.09	−1.09	0.01	0.00	0.01
19	1.97	1.97	0.03	0.00	0.03	3.66	3.66	0.03	0.00	0.03
20	2.42	2.42	0.05	0.00	0.05	5.84	5.84	0.07	0.00	0.07
21	2.43	2.41	0.04	−0.02	0.05	5.47	5.45	0.07	−0.02	0.07
22	2.30	2.30	0.03	0.00	0.03	3.81	3.81	0.04	0.00	0.04
23	1.98	1.98	0.03	0.00	0.03	3.43	3.43	0.03	0.00	0.03
24	1.78	1.78	0.03	0.00	0.03	4.42	4.43	0.05	0.00	0.05
25	1.61	1.60	0.02	0.00	0.02	−0.11	−0.11	0.01	0.00	0.01
26	2.15	2.15	0.02	0.00	0.02	−2.58	−2.58	0.02	0.00	0.02
27	1.60	1.61	0.02	0.00	0.02	0.91	0.91	0.01	0.00	0.01
28	1.58	1.58	0.02	0.00	0.02	−2.07	−2.07	0.02	0.00	0.02
29	2.46	2.46	0.03	0.00	0.03	2.28	2.27	0.03	0.00	0.03
30	2.45	2.46	0.02	0.01	0.02	0.41	0.41	0.02	0.00	0.02
31	1.76	1.76	0.03	0.00	0.03	−4.42	−4.42	0.04	0.00	0.04
32	1.88	1.88	0.05	0.00	0.05	−5.59	−5.59	0.08	−0.01	0.08
33	2.17	2.18	0.06	0.01	0.06	−6.47	−6.49	0.10	−0.02	0.10
34	2.47	2.48	0.04	0.00	0.04	−4.38	−4.38	0.05	0.00	0.05
35	1.94	1.93	0.03	−0.01	0.03	−4.87	−4.87	0.05	0.01	0.05
36	2.15	2.15	0.04	0.00	0.04	−4.47	−4.47	0.05	0.00	0.05
37	3.17	3.17	0.05	0.00	0.05	6.09	6.08	0.07	0.00	0.07
38	3.28	3.27	0.09	−0.01	0.09	8.27	8.25	0.17	−0.02	0.17
39	2.60	2.60	0.04	0.00	0.04	4.54	4.55	0.06	0.01	0.06
40	2.53	2.52	0.06	0.00	0.06	6.67	6.67	0.10	0.00	0.10
41	3.36	3.35	0.11	−0.01	0.11	9.82	9.78	0.25	−0.04	0.25
42	2.69	2.70	0.05	0.00	0.05	6.16	6.16	0.09	0.00	0.09
43	2.74	2.74	0.03	0.00	0.03	1.16	1.16	0.02	0.00	0.02
44	2.65	2.64	0.03	0.00	0.03	2.89	2.89	0.03	0.00	0.03
45	2.60	2.59	0.03	0.00	0.03	2.71	2.71	0.03	0.00	0.03
46	2.64	2.64	0.03	0.00	0.03	3.73	3.74	0.03	0.00	0.03
47	3.23	3.22	0.04	0.00	0.04	−1.71	−1.71	0.02	0.00	0.02
48	2.94	2.94	0.03	0.00	0.03	3.31	3.31	0.04	0.00	0.04
49	2.68	2.67	0.06	−0.01	0.06	−6.46	−6.44	0.10	0.02	0.10
50	2.77	2.76	0.07	−0.01	0.07	−7.12	−7.10	0.12	0.02	0.12
51	2.87	2.87	0.05	0.00	0.04	−5.50	−5.50	0.06	0.00	0.06
52	3.38	3.39	0.06	0.00	0.06	−5.92	−5.93	0.09	−0.01	0.09
53	2.60	2.60	0.08	0.00	0.08	−7.70	−7.70	0.17	0.00	0.17
54	3.46	3.45	0.06	−0.01	0.06	−5.83	−5.82	0.09	0.01	0.09

Simulation 2: By-Parameter Results

Table S6. Study 2 (10% Subset Size + 0.90 Threshold): Bias and RMSE by Parameter

Item	Parameter A				Parameter C			
	SE_{fis}	Mean SE_{xpd}	Bias	RMSE	SE_{fis}	Mean SE_{xpd}	Bias	RMSE
1	0.015	0.019	0.004	0.010	0.013	0.017	0.004	0.009
2	0.022	0.028	0.006	0.014	0.021	0.026	0.006	0.013
3	0.026	0.032	0.007	0.016	0.024	0.030	0.006	0.015
4	0.019	0.024	0.005	0.012	0.016	0.020	0.004	0.010
5	0.020	0.025	0.005	0.013	0.015	0.018	0.004	0.009
6	0.024	0.031	0.006	0.015	0.014	0.018	0.004	0.009
7	0.018	0.023	0.005	0.012	0.012	0.015	0.003	0.008
8	0.029	0.037	0.008	0.019	0.018	0.023	0.005	0.012
9	0.018	0.022	0.005	0.011	0.014	0.018	0.004	0.009
10	0.019	0.023	0.005	0.012	0.016	0.020	0.004	0.010
11	0.017	0.022	0.005	0.011	0.015	0.019	0.004	0.010
12	0.017	0.021	0.004	0.011	0.015	0.019	0.004	0.010

Table S7. Study 2 (20% Subset Size + 0.90 Threshold): Bias and RMSE by Parameter

Item	Parameter A				Parameter C			
	SE_{fis}	Mean SE_{xpd}	Bias	RMSE	SE_{fis}	Mean SE_{xpd}	Bias	RMSE
1	0.015	0.017	0.001	0.004	0.013	0.015	0.001	0.004
2	0.022	0.024	0.002	0.006	0.021	0.022	0.002	0.006
3	0.026	0.028	0.002	0.007	0.024	0.026	0.002	0.006
4	0.019	0.021	0.002	0.005	0.016	0.017	0.001	0.004
5	0.020	0.021	0.002	0.005	0.015	0.016	0.001	0.004
6	0.024	0.027	0.002	0.007	0.014	0.015	0.001	0.004
7	0.018	0.020	0.002	0.005	0.012	0.013	0.001	0.003
8	0.029	0.032	0.003	0.008	0.018	0.020	0.002	0.005
9	0.018	0.019	0.002	0.005	0.014	0.015	0.001	0.004
10	0.019	0.020	0.002	0.005	0.016	0.017	0.001	0.004
11	0.017	0.019	0.002	0.005	0.015	0.016	0.001	0.004
12	0.017	0.018	0.002	0.005	0.015	0.017	0.001	0.004

Table S8. Study 2 (30% Subset Size + 0.90 Threshold): Bias and RMSE by Parameter

Item	Parameter A				Parameter C			
	SE_{fis}	Mean SE_{xpd}	Bias	RMSE	SE_{fis}	Mean SE_{xpd}	Bias	RMSE
1	0.015	0.020	0.005	0.006	0.013	0.018	0.004	0.006
2	0.022	0.029	0.007	0.009	0.021	0.027	0.006	0.008
3	0.026	0.034	0.008	0.010	0.024	0.031	0.007	0.010
4	0.019	0.025	0.006	0.008	0.016	0.021	0.005	0.006
5	0.020	0.026	0.006	0.008	0.015	0.019	0.004	0.006
6	0.024	0.032	0.008	0.010	0.014	0.018	0.004	0.006
7	0.018	0.024	0.006	0.007	0.012	0.016	0.004	0.005
8	0.029	0.038	0.009	0.012	0.018	0.024	0.006	0.008
9	0.018	0.023	0.005	0.007	0.014	0.018	0.004	0.006
10	0.019	0.024	0.006	0.008	0.016	0.021	0.005	0.007
11	0.017	0.022	0.005	0.007	0.015	0.020	0.005	0.006
12	0.017	0.022	0.005	0.007	0.015	0.020	0.005	0.006

Table S9. Study 2 (50% Subset Size + 0.90 Threshold): Bias and RMSE by Parameter

Item	Parameter A				Parameter C			
	SE_{fis}	Mean SE_{xpd}	Bias	RMSE	SE_{fis}	Mean SE_{xpd}	Bias	RMSE
1	0.015	0.014	−0.001	0.002	0.013	0.013	−0.001	0.002
2	0.022	0.020	−0.001	0.002	0.021	0.019	−0.001	0.002
3	0.026	0.024	−0.002	0.003	0.024	0.022	−0.002	0.003
4	0.019	0.018	−0.001	0.002	0.016	0.015	−0.001	0.002
5	0.020	0.018	−0.001	0.002	0.015	0.014	−0.001	0.002
6	0.024	0.023	−0.002	0.003	0.014	0.013	−0.001	0.002
7	0.018	0.017	−0.001	0.002	0.012	0.011	−0.001	0.001
8	0.029	0.028	−0.002	0.003	0.018	0.017	−0.001	0.002
9	0.018	0.017	−0.001	0.002	0.014	0.013	−0.001	0.002
10	0.019	0.017	−0.001	0.002	0.016	0.015	−0.001	0.002
11	0.017	0.016	−0.001	0.002	0.015	0.014	−0.001	0.002
12	0.017	0.016	−0.001	0.002	0.015	0.014	−0.001	0.002

Table S10. Study 2 (100% Subset Size): Bias and RMSE by Parameter

Item	Parameter A				Parameter C			
	SE_{fis}	Mean SE_{xpd}	Bias	RMSE	SE_{fis}	Mean SE_{xpd}	Bias	RMSE
1	0.015	0.014	−0.001	0.001	0.013	0.012	−0.001	0.001
2	0.022	0.020	−0.002	0.002	0.021	0.019	−0.002	0.002
3	0.026	0.023	−0.002	0.002	0.024	0.022	−0.002	0.002
4	0.019	0.017	−0.002	0.002	0.016	0.014	−0.001	0.001
5	0.020	0.018	−0.002	0.002	0.015	0.013	−0.001	0.001
6	0.024	0.022	−0.002	0.002	0.014	0.013	−0.001	0.001
7	0.018	0.017	−0.002	0.002	0.012	0.011	−0.001	0.001
8	0.029	0.027	−0.003	0.003	0.018	0.017	−0.002	0.002
9	0.018	0.016	−0.002	0.002	0.014	0.013	−0.001	0.001
10	0.019	0.017	−0.002	0.002	0.016	0.015	−0.001	0.001
11	0.017	0.016	−0.002	0.002	0.015	0.014	−0.001	0.001
12	0.017	0.015	−0.002	0.002	0.015	0.014	−0.001	0.001
